# The effects of stimulated thyroglobulin/ thyroid-stimulating hormone ratio and N1b status on the efficacy of the first I-131 treatment in patients with differentiated thyroid cancer

**DOI:** 10.1371/journal.pone.0349850

**Published:** 2026-08-17

**Authors:** Supanida Mayurasakorn, Charoonsak Somboonporn, Daris Theerakulpisut

**Affiliations:** 1 Division of Nuclear Medicine, Department of Radiology, Faculty of Medicine, Khon Kaen University, Khon Kaen, Thailand; 2 National Cyclotron and PET Centre, Chulabhorn Hospital, Chulabhorn Royal Academy, Bangkok, Thailand; Universita degli Studi della Campania Luigi Vanvitelli, ITALY

## Abstract

**Background:**

Thyroglobulin (Tg) level is one of the well-known factors affecting the outcome of radioactive iodine treatment in differentiated thyroid cancer, though this tumor marker is affected by the thyroid-stimulating hormone (TSH) level. To negate this influence, there is an effort to use the stimulated thyroglobulin/ thyroid-stimulating hormone (sTg/TSH) ratio instead of stimulated thyroglobulin (sTg) level alone. N1b cervical lymph node metastasis is also another factor mentioned in literature. This study aims to demonstrate the significance of these two factors whether they affect the treatment outcome in differentiated thyroid cancer patients who received the first radioactive iodine treatment.

**Methods:**

This was a retrospective analytical observational study. The population were patients with differentiated thyroid cancer with an age of 18 years and above who received the first radioactive iodine treatment at our center from 1 January 2020–31 December 2023. The total population was 227 patients. Logistic regression analysis was done to determine the significant factors affecting the outcome of the first radioactive iodine treatment.

**Results:**

In 227 patients, the median value for sTg and sTg/TSH ratio were 9.4 ng/mL and 0.2, respectively. Multiple logistic regression found that sTg/TSH ratio and N1b status were not independent risk factors for non-excellent response after radioactive iodine treatment. The independent risk factors were male sex, intermediate and high risk of recurrence, stage II and sTg of more than or equal to 9.4 ng/mL. Receiver operating characteristic (ROC) analysis showed that sTg and sTg/TSH ratio had rather similar performance in differentiating between excellent and non-excellent response groups.

**Conclusion:**

sTg/TSH ratio and N1b cervical lymph node metastasis were not significant independent factors for non-excellent response after the first radioactive iodine treatment, which was possibly due to multicollinearity and small sample size. Independent risk factors for non-excellent response of the first radioiodine treatment were male sex, intermediate and high risk of recurrence, stage II and sTg of more than or equal to 9.4 ng/mL.

## Introduction

Differentiated thyroid cancer (DTC) is one of the common cancers encountered in clinical practice. According to the Global Cancer Statistics (GLOBOCAN) in 2020, thyroid cancer ranked 9th among cancers worldwide and is the most frequently encountered endocrine gland cancer [1]. The majority of cases are papillary thyroid carcinoma (PTC), accounting for over 90%, followed by follicular carcinoma at about 4%, and oncocytic thyroid carcinoma at approximately 2%. [2,3]

Although thyroid cancer currently has well-defined treatment guidelines, up to the endpoint of this study, following the American Thyroid Association (ATA) guidelines in 2015 [4]. There remains heterogeneity within each risk of recurrence group which results in different responses to treatment and disease course among patients classified in the same risk category.

Radioactive iodine (RAI) treatment is one of the important treatment modalities in DTC. Factors affecting the success of RAI treatment are diverse, but one well-known factor is the level of thyroglobulin (Tg), a glycoprotein produced specifically by the thyroid gland. However, thyroglobulin value can be affected by changes in thyroid-stimulating hormone (TSH) levels, which are regulated by the hypothalamic-pituitary-thyroid axis. To mitigate the impact of TSH on Tg measurement, the ratio of stimulated thyroglobulin to TSH (sTg/TSH ratio) has been proposed as a more accurate indicator [5–13].

Another important factor, often associated with unsuccessful RAI therapy and less studied, is lymph node metastasis in the lateral cervical region (N1b) [14–21].

This study aims to investigate the influence of sTg/TSH ratio and lateral cervical lymph node metastasis (N1b) on the treatment response of patients who have initial RAI therapy for thyroid cancer. Additional receiver operating characteristic (ROC) analysis for the optimal cut-off of sTg and sTg/ratio is also performed.

## Methods

### Patients

Approved by the Khon Kaen University Ethics Committee for Human Research (protocol number HE661275), this single-center, retrospective study had its informed consent requirement waived due to its design. The hospital director authorized access to patient data.

Studied population were histological-proven differentiated thyroid cancer (DTC) patients aged 18 years and older who received the first radioactive iodine (RAI) therapy at Division of Nuclear Medicine, Department of Radiology, Srinagarind Hospital, Faculty of Medicine, Khon Kaen University.

Initial ethics approval was granted on 13 June 2023 which allowed access to retrospective data from 1 January 2018–31 December 2022. After preliminary analysis of data in this period, it was found that data older than 31 December 2019 were not appropriate for analysis since the laboratory technique used at the time was radioimmunoassay which was later replaced by chemiluminescence immunoassay. The reference range of these two methods was different and could not be directly compared to each other. Therefore, an amendment request was made to the ethics committee to change the data range from 1 January 2020–31 December 2023. The amendment was approved on 7 April 2024. The researchers had access to the patient’s identifying information during the data collection process. Information which would lead to the identification of the patient, hospital number, in this case, was recorded in a separate file which could be only assessed by the researchers and used only for verification purposes. No information which would lead to identification of the patient was included in the final data set for analysis and discussion. The dataset in the supporting information was also completely anonymized.

Patients were excluded if no reported sTg, TSH and TgAb, no SPECT/CT of the neck with the post-treatment whole body scan (WBS), unclear staging or risk of recurrence, missing data of treatment response evaluation, receiving RAI more than 6 months post-thyroidectomy, having a TSH level less than 25 μIU/mL before treatment, using recombinant human thyrotropin (rhTSH) for preparation, initial TgAb level >30 IU/mL by electrochemiluminescence immunoassay (ECLIA), receiving other treatments before, or using radioimmunoassay (RIA) which was different from the method currently used in our center.

### Factors and outcome

Patients’ relevant clinical information was extracted from medical records including age at diagnosis, sex, surgical history, pathology report, staging according to AJCC/IUCC (8^th^ edition), sTg, TSH and TgAb level, radioactive iodine dosage, risk of recurrence and treatment response according to ATA 2015 guideline. Determination of nodal and distant metastasis status is based on tissue diagnosis from surgery or radioiodine-avid lesion from the post-treatment WBS.

Laboratory assay for sTg, TgAb and TSH were electrochemiluminescence immunoassay (Roche, Rotkreuz, Switzerland). Radioactive iodine treatment follows an institutional protocol. The dosage of radioactive iodine corresponds with ATA risk of recurrence category and tumor staging. A dosage of 30 mCi is prescribed for patients with low-risk of recurrence and T1 or T2 disease. A dosage of 100 mCi is prescribed for low-risk of recurrence and T3 disease. A dosage of 150 mCi is prescribe for patients with intermediate and high-risk patients as well as those with distant lung metastases and those with T4 disease. A dosage of 200 mCi is reserved for patients with bone metastases.

After radioactive iodine administration for 3–14 days, post-treatment WBS and SPECT/CT of the neck region, visualized from the base of the skull down to the upper chest, was performed using Discovery NM/CT 670 (GE Healthcare, IL, USA) or Discovery NM 830 (GE Healthcare, IL, USA). Image analysis was performed using the Xeleris program (GE Healthcare, IL, USA). Image interpretation was performed by an experienced nuclear medicine physician.

Between 6 and 18 months following radioactive iodine treatment, the treatment response was evaluated as the primary outcome of the study. The outcome of this study was a binary outcome, i.e., excellent and non-excellent response. The excellent response group includes patients who had no clinical, biochemical, or structural evidence of disease, as determined by negative imaging results and a sTg level of less than 1 ng/mL. While non-excellent response group included indeterminate response, biochemical incomplete response, and structural incomplete response according to the ATA guideline 2015.

### Statistical analysis

Patient characteristics were summarized using descriptive statistics. Continuous variables were reported as mean with standard deviation, if normally distributed, or median with interquartile range, if non-normally distributed. Categorical data was expressed as counts and percentages. Maximum and minimum value of the variables was also reported. Logistic regression was used to determine predictive factors of radioactive iodine treatment outcome. First, univariable logistic regression was performed on all predictors to screen for candidate variables. At this stage, predictors with a p-value of <0.2 was considered as a potential candidate and proceeded to be analyzed in the multivariable analysis. This liberal p-value threshold was chosen to avoid excluding potential important predictors which could turn out to be significant in the multivariable model. Subsequently, the selected variables were analyzed using multivariable logistic regression to determine the independent factors with a significant level of p-value <0.05 at this stage. Receiver Operating Characteristic (ROC) analysis was also performed to determine the optimal sTg and sTg/TSH ratio cut-offs in differentiating between excellent and non-excellent response groups.

In cases where extreme values were present, for example variables reported as exceeding the upper limit or falling below the lower limit in laboratory tests, such as sTg < 0.04 ng/mL, sTg > 500 ng/mL, or TSH > 100 µIU/mL, it was impossible to calculate the true numerical value of the sTg/TSH ratio. This was not uncommon in real-world clinical practice. The analysis was therefore performed in two ways: first, by excluding data from patients whose variable values exceeded the upper limit or fell below the lower limit of detection, i.e., sTg < 0.04 ng/mL, sTg > 500 ng/mL, or TSH > 100 µIU/mL; and second, by dividing the population into subgroups to cover all potential combinations of sTg and TSH results.

A total of seven subgroups were defined based on different combinations of sTg, TSH, and the sTg/TSH ratio, as follows. Subgroup 1 comprised patients with both sTg and TSH within reportable ranges and sTg/TSH ratio < mean or median. Subgroup 2 comprised patients with both sTg and TSH within reportable ranges and sTg/TSH ratio ≥ mean or median. Subgroup 3 included patients with TSH within a reportable range and sTg < 0.04 ng/mL. Subgroup 4 included patients with TSH within a reportable range and sTg > 500 ng/mL. Subgroup 5 included patients with TSH > 100 µIU/mL and sTg < 0.04 ng/mL. Subgroup 6 included patients with TSH > 100 µIU/mL and sTg > 500 ng/mL. Subgroup 7 included patients with TSH > 100 µIU/mL and sTg within a reportable range.

Each subgroup was analyzed independently using logistic regression. The subgroups were also further combined into two broader groups based on clinical perspective: a potential low Tg and low sTg/TSH ratio group, comprising Subgroups 1, 3, 5, and 7, and a potential high Tg and high sTg/TSH ratio group, comprising Subgroups 2, 4, and 6. These two broader groups were also analyzed by logistic regression.

Descriptive statistics and logistic regression were performed using STATA version 18 (StataCorp LLC, TX, USA). ROC analysis, which was analyzed using R Statistical Software (version 4.2.1; R Core Team), RStudio version 2023.12.1 + 402 “Ocean Storm” (RStudio, Boston, MA) and the R package, pROC. Power calculation was done using the R package, WebPower.

### Sample size considerations

All patient records that are available in the research data collection period which met the aforementioned inclusion and exclusion criteria were included in the analysis. To determine the adequacy of the final sample size, power calculation was done using the R package, WebPower. The statistical power was determined based on the difference of the prevalence of non-excellent response in cases with a positive versus negative key predictor of interest, and a significance level of 0.05.

## Results

The population included in the analysis was 227 subjects, among which 153 (67.4%) had non-excellent response outcome. Among patients with N1b node status the non-excellent outcome was 82%, as compared with 60% in those without N1b lymph nodes. This resulted in a statistical power of approximately 89%. Detailed characteristics of the cohort were presented in S1 Table. Since the variable exhibited a non-normal distribution, the median was used instead of the mean. The majority of the population was female (80%), with a median age of 51 years. The predominant histological type was papillary thyroid carcinoma (79%), followed by follicular thyroid carcinoma (10%). Approximately half of the patients had lymph node metastasis. Among those with lymph node spread, more than half (63%) involved the lateral cervical lymph node group (N1b). Only a minority of patients (6.6%) had distant metastasis. Most of the patients had stage I cancer (68%). About one-third (33%) of patients achieved an excellent response to treatment. Regarding the laboratory values, the median sTg, median TSH and median sTg/TSH ratio was 9.4 ng/mL, 71.5 µIU/mL and 0.2, respectively. These values were used as a cut-off for dividing aforementioned sTg/TSH ratio and TSH subgroups.

Multivariable logistic regression analysis was performed on 227 participants. Model performance was evaluated using the Akaike Information Criterion (AIC = 223.6) and Hosmer-Lemeshow goodness-of-fit test (χ²(8) = 8.62, p = 0.376), confirming adequate model fit. Logistic regression analysis results of sTg/TSH ratio, lateral cervical lymph node metastasis and other significant variables were shown in Table I, the detailed results were shown in S2 Table.

Since a total of 5 variables were included in the multivariable logistic regression analysis, at least 50 events were needed according to the 10-events per variable rule. In this study, there are a total of 153 events, which is adequate for a reliable regression model (Table 1).

**Table 1:** Logistic regression analysis results.

Variable	Total cases (N = 227)	Univariable		Multivariable	
		OR (95% CI)	P-value	Adjusted OR (95% CI)	P-value
sTg/TSH ratio (IQR)	0.2 (0.06,0.68)	17.97 (2.68,120.6)	0.003		
sTg (IQR)	9.37 (3.77,34.5)	1.04 (1.02,1.06)	<0.001		
sTg < 9.4	107	Ref.			
sTg ≥ 9.4 (including sTg > 500)	120	6.91 (3.65,13.08)	<0.001	5.29 (2.6,10.75)	<0.001
**Cervical node metastasis location** N1a	36	Ref.			
N1b	74	0.59 (0.18,1.95)	0.383		
N1x	8	0.38 (0.06,2.53)	0.314		
Male	46	3.27 (1.39,7.73)	0.007	4.78 (1.76,12.96)	0.002
Stage II (compared with stage I)	62	4.19 (1.93,9.1)	<0.001	2.63 (1.09,6.35)	0.032
Intermediate risk of recurrence (compared with low risk)	97	3.4 (1.78,6.51)	<0.001	2.92 (1.39,6.12)	0.005
High risk of recurrence (compared with low risk)	61	12.64 (4.8,33.31)	<0.001	5.86 (2.01, 17.03)	0.001
age (IQR)	51 (38,61)	1.02 (1.00,1.04)	0.018		
Multifocality	94	1.97 (1.09,3.54)	0.024		
Gross extrathyroidal extension (compared with no ETE)	18	5.42 (1.18,24.81)	0.029		
Minimal extrathyroidal extension (compared with no ETE)	34	1.88 (0.82, 4.63)	0.148		
Unknown extrathyroidal extension (compared with no ETE)	71	1.61 (0.85, 3.10)	0.145		
Positive surgical margin	41	4.45 (1.66,11.93)	0.003		
Presence of cervical lymph node metastasis	118	5.31 (2.86,9.85)	<0.001		
I-131 150 mCi	143	3.71 (2.05,6.71)	<0.001		
**sTg/TSH ratio subgroup** Subgroup 1	62	Ref.			
Subgroup 2	64	4.71 (1.98,11.2)	<0.001		
Subgroup 4	7	NA			
Subgroup 6	4	NA			
Subgroup 7	90	1.06 (0.55,2.03)	0.871		
sTg/TSH ratio subgroup 1, 3, 5, 7	152	Ref.			
sTg/TSH ratio subgroup 2, 4, 6	75	5.48 (2.54,11.8)	<0.001		
**Subgroup 7: TSH > 100 µIU/mL and sTg have a reportable value** Tg < 9.4	52	Ref.			
Tg ≥ 9.4	38	5.11 (1.97,13.28)	0.001		

N1x; nodal metastasis that could not be clearly identified whether it was stage N1a or N1b, NA; not applicable, Ref.; reference.

**Fig 1:**
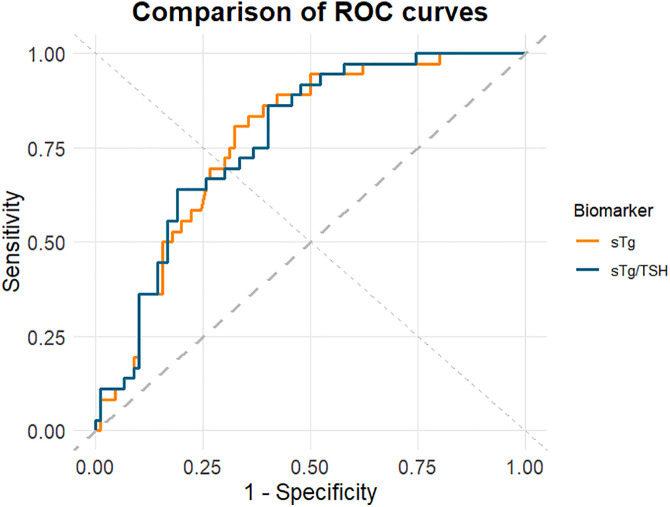
The ROC analysis of sTg and the sTg/TSH ratio. This demonstrated the ability of both variables to differentiate between the excellent and non-excellent response groups. The two graphs were found to be very similar, with the yellow line representing the sTg value and the green line representing the sTg/TSH ratio, respectively.

Comparing the ROC curves of sTg and sTg/TSH ratio, ROC curves of both variables were very similar, and their respective area under the curve (AUC) values were also very close (Fig 1). The AUC and 95% confidence interval (95%CI) for sTg and the sTg/TSH ratio were 0.7731 (95%CI:0.6899–0.8564) and 0.7725 (95%CI:0.6894–0.8556), respectively. DeLong’s test yielded a P-value of 0.729 which indicates no significant difference between the area under the curves of the two ROCs.

**Fig 2:**
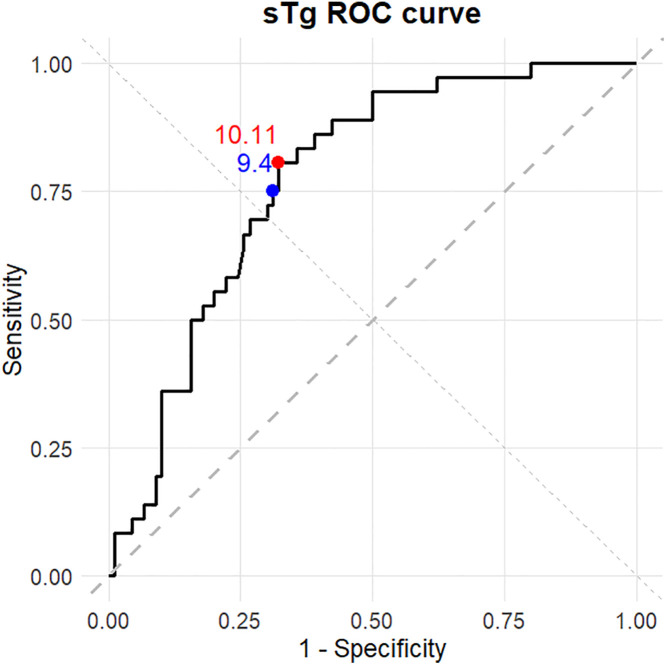
A comparison of the positions of different sTg cut-off values on the ROC curve. The blue dot represented the cut-off value derived from the study’s median, and the red dot represented the cut-off value derived from the Youden index.

**Fig 3:**
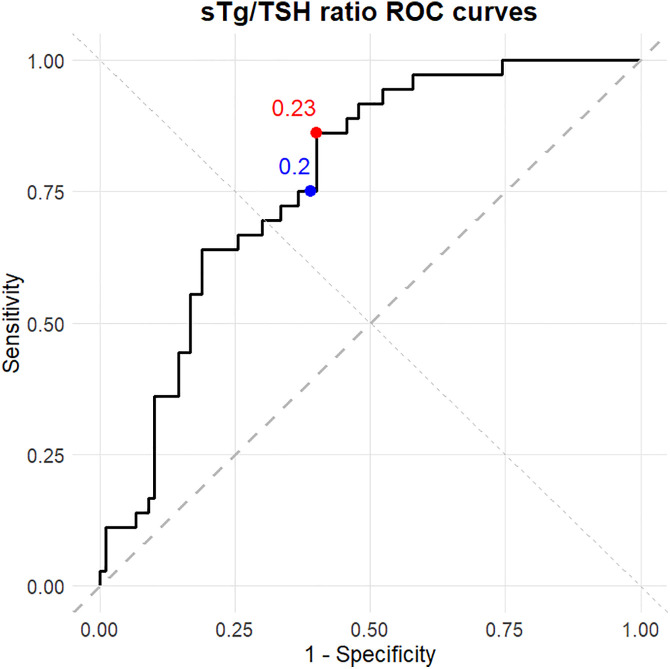
A comparison of the positions of different sTg/TSH ratio cut-off values on the ROC curve. The blue dot represented the cut-off value derived from the study’s median, and the red dot represented the cut-off value derived from the Youden index.

For the assessment of the optimal cut-off values for sTg and sTg/TSH ratio, two methods were used: First, the cut-off was derived from the median of the study, and second, it was derived from the value with the highest Youden index. The location of these cut-off points on the ROC curve was shown in Figs 2 and 3. The numerical cut-off values, along with the corresponding sensitivity and specificity, were presented in Table 2.

**Table 2:** Cut-off values of sTg and sTg/TSH ratio from the median and Youden index.

Variables and cut-offs	Sensitivity	Specificity
sTg 9.4 ng/mL (median)	0.65	0.77
sTg 10.1 ng/mL (Youden index)	0.81	0.67
sTg/TSH ratio 0.2 (median)	0.61	0.75
sTg/TSH ratio 0.23 (Youden index)	0.86	0.60

The cut-off values by two methods were very close. However, there might be some differences in the diagnostic performance due to a trade-off between sensitivity and specificity resulting from the selection of different cut-off values.

## Discussion

The primary objective of this study was to determine whether the sTg/TSH ratio and lateral cervical lymph node metastasis (N1b) were significant factors affecting the outcome of the first radioactive iodine treatment in patients with differentiated thyroid cancer. The optimal cut-off for sTg and the sTg/TSH ratio to differentiate between the excellent and non-excellent response groups were also analyzed using ROC analysis.

From the logistic regression analysis, the independent risk factors for developing a non-excellent response to treatment were: male sex, ATA intermediate and high risk of recurrence, stage II cancer, and an sTg value greater than or equal to 9.4 ng/mL.

Regarding the male sex and ATA intermediate and high risk of recurrence factors, these findings were consistent with previous studies [9,15,20]. The ATA guideline 2015 [4] also states that a higher risk of recurrence was associated with an increased risk of structural persistent/recurrent disease.

Generally, as the cancer stage increased, a poor treatment outcome was more likely to be observed. This was consistent with the results of this study, which found that stage II cancer significantly affected treatment outcome negatively compared to stage I. Stage III cancer, however, was not found to be a factor causing an increased poor treatment outcome. This might be due to the low number of patients in stage III, resulting in insufficient statistical power. For stage IV cancer, the odds ratio could not be calculated because the rate of the event of interest, a poor treatment outcome, was 100%.

An sTg ≥ 9.4 ng/mL, including a group with sTg > 500 ng/mL, was associated with a non-excellent response, serving as an independent risk factor (adjusted OR=5.29; 95%CI:2.6–10.75; p < 0.001). This finding was true even within the subgroup 7 (TSH > 100 µIU/mL with reportable sTg value) with crude OR of 5.11 (95%CI:1.97–13.28, p-value 0.001). The finding that a high sTg value led to a poor treatment outcome was consistent with several previous literature.

Regarding the sTg/TSH ratio, a high ratio was found to have a crude OR for a non-excellent response of 17.97 (95%CI:2.68–120.58, p-value = 0.003). However, it lost significance in the multivariable logistic regression analysis. The loss of significance and the wide confidence interval might be due to the multicollinearity among the studied variables. Nevertheless, it was found that subgroup 2 (sTg/TSH ratio ≥0.2) and other subgroups where the sTg/TSH ratio was likely to be high, such as subgroup 4 (TSH reportable and Tg > 500 ng/mL) and subgroup 6 (TSH > 100 µIU/mL and Tg > 500 ng/mL), all showed significance for a poor treatment outcome in univariable logistic regression analysis.

Other variables which were found to be significant in the univariable logistic regression, but not significant in the multivariable logistic regression analysis might be partially explained by the multicollinearity. For example, the correlation of gross ETE with having a positive margin and being classified into the high risk of recurrence group, cervical node metastasis with being classified into the intermediate risk of recurrence group. For the radioactive iodine dosage, patients receiving higher radioiodine dosage often had a poorer prognosis; this is likely due to their baseline disease severity rather than the treatment itself. The presence or absence of cervical node metastasis was not the only factor that might affect treatment outcome. The characteristics of the lymph node itself, such as size and number, were also important. For age, although it was generally believed that increasing age was associated with worse disease progression, some studies had found that age was not an independent risk factor for a poor treatment outcome. Thamnirat et al. in 2015 [22] in 256 patients found that age ≤ 45 years was not associated with disease-free status in multivariate analysis. Additionally, a study by Ganly et al. in 2015 [23] involving 3,664 thyroid cancer patients found that when age was considered as a continuous variable affecting disease-specific survival, the adjusted hazard ratio (aHR) was 1.076 (95%CI:1.06–1.1, p-value<0.001). The confidence interval was close to 1, the null hypothesis, thus studies with smaller populations, such as this study and Thamnirat et al., might not have sufficient power enough to find age as a significant factor.

Lateral cervical lymph node metastasis (N1b) was found to be non-significant for the development of a non-excellent response in this study. This lack of significance was possibly due to the insufficient sample size of the population with this specific factor. For other lymph node characteristics such as numbers, size, and extranodal extension, these factors were analyzed in the univariate analysis, as shown in S2 Table. None of the factors showed significance in predicting treatment outcome. Due to the fact that some patients were referred from other hospitals, completed details of lymph node characteristics could not be obtained for some cases.

The ability of sTg and the sTg/TSH ratio to differentiate between patients with excellent and non-excellent response outcomes, as shown by the ROC analysis, was found to be rather similar based on the shape of the graph, AUC values and also the cut-off values from the median and Youden index were rather close, at 9.4 and 10.1 ng/dL, respectively. However, it is notable that this minimal shift in the cutoff point causes the sensitivities and specificities to be “flipped”, with the cutoff of 9.4 ng/mL having a sensitivity of 65% and a specificity of 77%, while moving the cutoff to 10.1 ng/dL results in a higher sensitivity of 81% and a lower specificity of 67%. This can be explained by “datapoint clumping” with many of the patients with excellent response, and non-excellent response having the sTg around these threshold boundaries. That being said, in our opinion, a cutoff of 10.1 ng/dL based on the Youden index would be more appropriate as it gives a higher sensitivity and an acceptable specificity. This cutoff is also similar to the findings of a large study by Webb et al. in 2012 [24], which was a meta-analysis of 3,947 patients. That study proposed that a pre-ablation Tg value less than 10 ng/mL was a predictor with a high negative predictive value for determining the disease-free status of patients. As for the sTg/TSH ratio, a similar phenomenon was found when analyzing the cutoff values. Moreover, the cutoff of 0.23 based on the Youden index yielded a sensitivity of 86% at the expense of a lower specificity of 60% compared with sTg. Therefore, the sTg/TSH ratio does not reveal superiority to sTg based on these current findings.

There were several limitations of this study. First, this study has been conducted before the publication of new ATA guideline for differentiated thyroid cancer in 2025. Patients treated at our center were managed based on the now superseded ATA 2015 guidelines, thus there might be some difference in risk stratification and management of the patient. Second, due to retrospective single-center design of the study, this might lead to incomplete data for some variables, limitation in control of confounders and the data should be considered before applying to other population. Third, the nature of lymph node metastasis in thyroid cancer could be difficult to diagnose in some situations because nodal dissection is not routinely done in thyroid cancer and nodal metastasis might be small and had minimal RAI uptake, resulting in limited sample size and potential false negative. Fourth, certain factors with insufficient numbers of patients and presence of a relatively high degree of multicollinearity among the variables meant that some variables might be found to be non-significant in the statistical analysis. Lastly, due to the rather indolent disease course of thyroid cancer, limited follow-up time might not truly reflect the long-term outcome of the patients, thus longer follow-up time was encouraged in further studies.

## Conclusion

The sTg/TSH ratio and the presence of lateral cervical lymph node metastasis (N1b) were not independent risk factors for a non-excellent response in this study, which could be due to a relatively small sample size in the context of the presence of multicollinearity with other well-established predictors such as sTg.

The independent risk factors for developing a non-excellent response to radioactive iodine treatment in patients with differentiated thyroid cancer were: male sex, intermediate and high risk of recurrence according to ATA guideline 2015, Stage II cancer, and an sTg value greater than or equal to 9.4 ng/mL.

## Supporting information

S1 Table Detailed clinical characteristics of the cohort. (DOCX)

S2 Table Detailed results from logistic regression analysis for factors associated with non-excellent response outcome after I-131 treatment. NA; not applicable, Ref.; reference.
(DOCX)

S3 Dataset Anonymized dataset. (XLSX)
