# Supplementary material for: The effects of stimulated thyroglobulin/ thyroid-stimulating hormone ratio and N1b status on the efficacy of the first I-131 treatment in patients with differentiated thyroid cancer
Source: PLoS One. 2026 Aug 17;21(8):e0349850. doi: 10.1371/journal.pone.0349850 (PMC13480613; doi:10.1371/journal.pone.0349850)
Supplement: Supplementary file 1 [file pone.0349850.s001.docx]

**S1 Table. Detailed clinical characteristics of the cohort**

| **Characteristic** | **Value** |
| --- | --- |
| **Age (years)**     Median     Min – Max     Interquartile range | 51  18 – 87  38 – 61 |
| **Sex (number, %)**     Female     Male | 181 (79.74)  46 (20.26) |
| **Surgical type (number, %)**     Total thyroidectomy     Near total thyroidectomy     Completion thyroidectomy | 140 (61.67)  3 (11.11)  87 (37.00) |
| **Neck dissection (number, %)**     Yes     No | 55 (24.23)  172 (75.77) |
| **Incomplete tumor resection (number, %)**     Yes     No | 4 (1.76)  223 (98.24) |
| **sTg (ng/mL)**     Median     Min – Max     Interquartile range | 9.37  0.06 – 3,513  3.77 – 34.47 |
| **sTg group by range (number, %)**     < Median     ≥ Median and ≤ 500 ng/mL     > 500 ng/mL | 107 (47.14)  109 (48.02)  11 (4.85) |

**S1 Table. Detailed clinical characteristics of the cohort (continue)**

| **Characteristic** | **Value** |
| --- | --- |
| **TSH (µIU/mL)**     Median     Min – Max     Interquartile range | 71.5  25.1 – 173.0  55.0 – 85.8 |
| **TSH group by upper limit (number, %)**     Within reportable range (≤100 µIU/mL)     Exceeding reportable range (>100 µIU/mL) | 133 (58.59)  94 (41.41) |
| **TSH group by range (number, %)**     < Median     ≥ Median and ≤ 100 µIU/mL     > 100 µIU/mL | 66 (29.07)  67 (29.52)  94 (41.41) |
| **sTg/TSH ratio (ng/µIU)**     Median     Min – Max     Interquartile range | 0.2  0.001 **–** 73.65  0.06 **–** 0.68 |
| **sTg/TSH ratio subgroup (number, %)**     Subgroup 1: sTg/TSH ratio < Median     Subgroup 2: sTg/TSH ratio ≥ Median     Subgroup 3: TSH has a reportable value and sTg<0.04 ng/mL     Subgroup 4: TSH has a reportable value and sTg>500 ng/mL     Subgroup 5: TSH >100 µIU/mL, sTg<0.04 ng/mL     Subgroup 6: TSH >100 µIU/mL, sTg>500 ng/mL     Subgroup 7: TSH >100 µIU/mL and sTg has a reportable value | 62 (27.31)  64 (28.19)  0 (0.00)  7 (3.08)  0 (0.00)  4 (1.76)  90 (39.65) |
| **Subgroup 7: TSH >100 µIU/mL and sTg has a reportable value  (number/total, %)**     sTg <9.4 ng/mL     sTg ≥9.4 ng/mL | 52/90 (57.78)  38/90 (42.22) |

**S1 Table. Detailed clinical characteristics of the cohort (continue)**

| **Characteristic** | **Value** |
| --- | --- |
| **Focality (number, %)**     Unifocal     Multifocal     Not reported | 131 (57.71)  94 (41.41)  2 (0.88) |
| **Histology of thyroid carcinoma (number, %)**     PTC     PTC with unfavorable variant     FTC     Widely invasive FTC     Oncocytic carcinoma     Poorly differentiated carcinoma     Others   - Mixed PTC and FTC - Mixed PTC with unfavorable variant with oncocytic carcinoma - Mixed FTC and oncocytic carcinoma - Mixed PDTC and PTC with unfavorable subtypes - Mixed PDTC and FTC - Well-differentiated thyroid carcinoma (NOS) | 179 (78.85)  2 (0.88)  24 (10.57)  1 (0.44)  9 (3.96)  2 (0.88)  5 (2.20)  1 (0.44)  1 (0.44)  1 (0.44)  1 (0.44)  1 (0.44) |
| **Unfavorable histology (number, %)**     Yes     No | 18 (7.93)  209 (92.07) |
| **Largest tumor size (cm)**     Median     Min – Max     Interquartile range | 2.7  0.1 **–** 11  1.5 **–** 4 |

**S1 Table. Detailed clinical characteristics of the cohort (continue)**

| **Characteristic** | **Value** |
| --- | --- |
| **Extrathyroidal extension (ETE) (number, %)**     No     Minimal ETE     Gross ETE     Not reported | 104 (45.81)  34 (14.98)  18 (7.93)  71 (31.28) |
| **Lymphovascular invasion (LVSI) (number, %)**     Absent     Not reported     Present   - Present ≤ 4 foci - Present > 4 foci - Present, unknown focus | 119 (52.42)  14 (6.17)  94 (41.41)  16/94 (17.02)  18/94 (19.15)  60/94 (63.83) |
| **Surgical margin (number, %)**     Positive     Negative     Not reported | 41 (18.06)  165 (72.69)  21 (9.25) |
| **Cervical lymph node metastasis (number, %)**     No     Yes   - N1a - N1b - N1x | 109 (48.02)  118 (51.98)  36/118 (30.51)  74/118 (62.71)  8/118 (6.78) |
| **Number of cervical lymph node metastasis (number, %)**     Median     Min – Max     Interquartile range | 2  1 **–** 26  1 **–** 7 |

**S1 Table. Detailed clinical characteristics of the cohort (continue)**

| **Characteristic** | **Value** |
| --- | --- |
| **Largest cervical lymph node metastasis size (cm)**     Median     Min – Max     Interquartile range | 1  0.2 **–** 6.6  0.5 **–** 2 |
| **Extranodal extension (ENE) (number, %)**     No     Yes | 100/118 (84.75)  18/118 (15.25) |
| **Distant metastasis (number, %)**     Yes     No | 15 (6.61)  212 (93.39) |
| **AJCC 8th TNM staging (number, %)**     Stage I     Stage II     Stage III     Stage IVA     Stage IVB | 154 (67.84)  62 (27.31)  4 (1.76)  0 (0.00)  7 (3.08) |
| **Iodine-131 dosage (number, %)**     30 mCi     100 mCi     150 mCi     200 mCi | 79 (34.80)  3 (1.32)  143 (63.00)  2 (0.88) |
| **Post-radioiodine therapy ATA risk of recurrence**  **(number, %)**     Low     Intermediate     High | 69 (30.40)  97 (42.73)  61 (26.87) |
| **Treatment response (number, %)**     Excellent     Non-excellent | 74 (32.60)  153 (67.40) |
