# Supplementary material for: The effects of stimulated thyroglobulin/ thyroid-stimulating hormone ratio and N1b status on the efficacy of the first I-131 treatment in patients with differentiated thyroid cancer
Source: PLoS One. 2026 Aug 17;21(8):e0349850. doi: 10.1371/journal.pone.0349850 (PMC13480613; doi:10.1371/journal.pone.0349850)
Supplement: Supplementary file 2 [file pone.0349850.s002.docx]

**S2 Table. Detailed results from logistic regression analysis for factors associated with non-excellent response outcome after I-131 treatment.** NA; not applicable, Ref.; reference.

| **Variable** | **Total cases** **(N = 227)** | **Cases with excellent response outcome** **(n=74)** | **Cases with non-excellent response outcome** **(n=153)** | **Univariable** | | **Multivariable** | | |
| --- | --- | --- | --- | --- | --- | --- | --- | --- |
|  |  |  |  | **OR (95% CI)** | **P-value** | | **Adjusted OR** **(95% CI)** | **P-value** |
| **Age** |  |  |  |  |  |  | |  |
| Median (IQR) | 51  (38,61) | 48  (33,55) | 52  (40,63) | 1.02  (1.00,1.04) | 0.018 |  | |  |
| **Sex** |  |  |  |  |  |  | |  |
| Female | 181  (100%) | 67  (37.0%) | 114  (63%) | Ref. |  |  | |  |
| Male | 46  (100%) | 7  (15.2%) | 39  (84.8%) | 3.27  (1.39,7.73) | 0.007 | 4.78  (1.76,12.96) | | 0.002 |
| **Incomplete tumor resection** | | | | | | | | |
| No | 223  (100%) | 74  (33.2%) | 149  (66.8%) | NA | NA |  | |  |
| Yes | 4  (100%) | 0  (0%) | 4  (100%) |  |  |  | |  |
| **Tumor size** |  |  |  |  |  |  | |  |
| Median (IQR) | 2.7  (1.5,4) | 2.55  (1.4,3.7) | 2.9  (1.8,4) | 1.18  (0.99,1.39) | 0.058 |  | |  |
| **Focality** |  |  |  |  |  |  | |  |
| Unifocality | 131  (100%) | 51  (38.9%) | 80  (61%) | Ref. |  |  | |  |
| Multifocality | 94  (100%) | 23  (24.5%) | 71  (75.5%) | 1.97  (1.09,3.54) | 0.024 |  | |  |
| **PTC** |  |  |  |  |  |  | |  |
| Others | 48  (100%) | 19  (40%) | 29  (60%) | Ref. |  |  | |  |
| PTC | 179  (100%) | 55  (30.7%) | 124  (69.3%) | 1.48  (0.76,2.86) | 0.247 |  | |  |

**S2 Table. Detailed results from logistic regression analysis for factors associated with non-excellent response outcome after I-131 treatment.** NA; not applicable, Ref.; reference. (continue)

| **Variable** | **Total cases (N = 227)** | **Cases with excellent response outcome (n=74)** | **Cases with non-excellent response outcome (n=153)** | **Univariable** | | **Multivariable** | |
| --- | --- | --- | --- | --- | --- | --- | --- |
|  |  |  |  | **OR (95% CI)** | **P-value** | **Adjusted OR (95% CI)** | **P-value** |
| **FTC** |  |  |  |  |  |  |  |
| Others | 203  (100%) | 62  (30.5%) | 141  (69.5%) | Ref. |  |  |  |
| FTC | 24  (100%) | 12  (50%) | 12  (50%) | 0.44  (0.19,1.03) | 0.059 |  |  |
| **Mixed PTC and FTC** | |  |  |  |  |  |  |
| Others | 222  (100%) | 72  (32.4%) | 150  (67.6%) | Ref. |  |  |  |
| Mixed PTC and FTC | 5  (100%) | 2  (40%) | 3  (60%) | 0.72  (0.12,4.4) | 0.722 |  |  |
| **Oncocytic carcinoma** | | | | | | | |
| Others | 218  (100%) | 71  (32.6%) | 147  (67.4%) | Ref. |  |  |  |
| Oncocytic carcinoma | 9  (100%) | 3  (33.3%) | 6  (66.67%) | 0.97  (0.23,3.97) | 0.962 |  |  |
| **ETE** |  |  |  |  |  |  |  |
| No ETE | 104  (100%) | 42  (40.4%) | 62  (59.6%) | Ref. |  |  |  |
| minimal ETE | 34  (100%) | 9  (26.5%) | 25  (73.5%) | 1.88  (0.8,4.43) | 0.148 |  |  |
| Gross ETE | 18  (100%) | 2  (11.1%) | 16  (88.9%) | 5.42  (1.18,24.81) | 0.029 |  |  |
| **LVSI** |  |  |  |  |  |  |  |
| No LVSI | 119  (100%) | 45  (37.8%) | 74  (62.2%) | Ref. |  |  |  |
| LVSI | 94  (100%) | 24  (25.5%) | 70  (74.5%) | 1.77  (0.98,3.21) | 0.058 |  |  |

**S2 Table. Detailed results from logistic regression analysis for factors associated with non-excellent response outcome after I-131 treatment.** NA; not applicable, Ref.; reference. (continue)

| **Variable** | **Total cases** **(N = 227)** | **Cases with excellent response outcome** **(n=74)** | **Cases with non-excellent response outcome** **(n=153)** | **Univariable** | | | **Multivariable** | | |
| --- | --- | --- | --- | --- | --- | --- | --- | --- | --- |
|  |  |  |  | **OR** **(95% CI)** | **P-value** | | **Adjusted OR** **(95% CI)** | | **P-value** |
| **Presence of LVSI** | | | | | | | | | |
| ≤4 foci | 16  (100%) | 6  (37.5%) | 10  (62.5%) | Ref. |  |  | |  | |
| >4 foci | 18  (100%) | 8  (44.4%) | 10  (55.5%) | 0.75  (0.19,2.96) | 0.682 |  | |  | |
| **Positive surgical margin** | | | | | | | | | |
| Negative | 165  (100%) | 63  (38.2%) | 102  (61.8%) | Ref. |  |  | |  | |
| Positive | 41  (100%) | 5  (12.2%) | 36  (87.8%) | 4.45  (1.66,11.93) | 0.003 |  | |  | |
| **Cervical lymph node metastasis** | | | | | | | | | |
| No | 109  (100%) | 55  (50.5%) | 54  (49.5%) | Ref. |  |  | |  | |
| Yes | 118  (100%) | 19  (16%) | 99  (83.9%) | 5.31  (2.86,9.85) | <0.001 |  | |  | |
| **Cervical lymph node metastasis location** | | | | | | | | | |
| N1a | 36  (100%) | 4  (11.1%) | 32  (88.9%) | Ref. |  |  | |  | |
| N1b | 74  (100%) | 13  (17.6%) | 61  (82.4%) | 0.59  (0.18,1.95) | 0.383 |  | |  | |
| N1x | 8  (100%) | 2  (25%) | 6  (75%) | 0.38  (0.06,2.53) | 0.314 |  | |  | |
| **Number of lymph node metastasis** | | | | | | | | | |
| Median (IQR) | 2  (1,7) | 2  (1,2.5) | 3  (1, 9) | 1.18  (0.95,1.46) | 0.125 |  | |  | |

**S2 Table. Detailed results from logistic regression analysis for factors associated with non-excellent response outcome after I-131 treatment.** NA; not applicable, Ref.; reference. (continue)

| **Variable** | **Total cases** **(N = 227)** | **Cases with excellent response outcome** **(n=74)** | **Cases with non-excellent response outcome** **(n=153)** | **Univariable** | | **Multivariable** | |
| --- | --- | --- | --- | --- | --- | --- | --- |
|  |  |  |  | **OR** **(95% CI)** | **P-value** | **Adjusted OR** **(95% CI)** | **P-value** |
| **Largest cervical lymph node metastasis size** | | | | | | | |
| Median (IQR) | 1  (0.5,2) | 0.5  (0.2,0.5) | 1.1  (0.5,2.1) | 2.77  (0.83,9.28) | 0.098 |  |  |
| **Extranodal extension** | | | | | | | |
| No | 100  (100%) | 18  (18%) | 82  (82%) | Ref. |  |  |  |
| Yes | 18  (100%) | 1  (5.6%) | 17  (94.4%) | 3.73  (0.47,29.88) | 0.215 |  |  |
| **AJCC 8th TNM staging** | | | | | | | |
| Stage I | 154  (100%) | 64  (41.6%) | 90  (58.4%) | Ref. |  |  |  |
| Stage II | 62  (100%) | 9  (14.5%) | 53  (85.5%) | 4.19  (1.93,9.1) | <0.001 | 2.63  (1.09,6.35) | 0.032 |
| Stage III | 4  (100%) | 1  (25%) | 3  (75%) | 2.13  (0.22,20.98) | 0.516 |  |  |
| Stage IVB | 7  (100%) | 0  (0%) | 7  (100%) | *OR cannot be calculated, due to the event of interest having a 100% chance to occur.* | | | |
| **I-131 dosage** |  |  |  |  |  |  |  |
| 30 mCi | 79  (100%) | 40  (50.6%) | 39  (49.4%) | Ref. |  |  |  |
| 100 mCi | 3  (100%) | 2  (66.7%) | 1  (3%) | 0.51  (0.04,5.89) | 0.592 |  |  |
| 150 mCi | 143  (100%) | 31  (21.7%) | 112  (78.3%) | 3.71  (2.05,6.71) | <0.001 |  |  |
| 200 mCi | 2  (100%) | 1  (50%) | 1  (50%) | 1.03  (0.06,16.98) | 0.986 |  |  |

**S2 Table. Detailed results from logistic regression analysis for factors associated with non-excellent response outcome after I-131 treatment.** NA; not applicable, Ref.; reference. (continue)

| **Variable** | | **Total cases** **(N = 227)** | | **Cases with excellent response outcome** **(n=74)** | | **Cases with non-excellent response outcome** **(n=153)** | | **Univariable** | | | | | **Multivariable** | | |  |
| --- | --- | --- | --- | --- | --- | --- | --- | --- | --- | --- | --- | --- | --- | --- | --- | --- |
|  |  |  |  |  |  |  |  | **OR** **(95% CI)** | | **P-value** | | | **Adjusted OR** **(95% CI)** | **P-value** | |  |
|  | **Post-radioiodine therapy ATA risk of recurrence** | | | | | | | | | | | | | | |  |
|  | Low | | 69  (100%) | | 40  (58%) | | 29  (42%) | | Ref. | |  |  | | |  | |
|  | Intermediate | | 97  (100%) | | 28  (29%) | | 69  (71%) | | 3.4  (1.78,6.51) | | <0.001 | 2.92  (1.39,6.12) | | | 0.005 | |
|  | High | | 61  (100%) | | 6  (9.8%) | | 55  (90.2%) | | 12.64  (4.8,33.31) | | <0.001 | 5.86  (2.01, 17.03) | | | 0.001 | |
|  | **sTg** | |  | |  | |  | |  | |  |  | | |  | |
|  | Median (IQR) | | 9.37  (3.77,34.5) | | 4.67  (2.18,8.56) | | 15.13  (6.06, 53) | | 1.04  (1.02,1.06) | | <0.001 |  | | |  | |
|  | **TSH** | |  | |  | |  | |  | |  |  | | |  | |
|  | Median (IQR) | | 71.5  (55,85.8) | | 77.5  (59.3,88.15) | | 70.4  (53.9,85.3) | | 0.99  (0.97,1.00) | | 0.085 |  | | |  | |
|  | **sTg/TSH ratio** | | | | | | | | | | | | |  | |  |
|  | Median (IQR) | | 0.2  (0.06,0.68) | | 0.06  (0.03,0.19) | | 0.43  (0.1,1.38) | | 17.97  (2.68,120.6) | | 0.003 |  | | |  | |
|  | **sTg (median sTg 9.4 ng/mL)** | | | | | | | | | | | | | | |  |
|  | sTg <Median | | 107 (100%) | | 57 (53%) | | 50 (47%) | | Ref. | |  |  | | |  | |
|  | sTg ≥Median  (including sTg >500) | | 120 (100%) | | 17 (14%) | | 103 (86%) | | 6.91 (3.65,13.08) | | <0.001 | 5.29 (2.6,10.75) | | | <0.001 | |

**S2 Table. Detailed results from logistic regression analysis for factors associated with non-excellent response outcome after I-131 treatment.** NA; not applicable, Ref.; reference. (continue)

| **Variable** | **Total cases (N = 227)** | **Cases with excellent response outcome (n=74)** | **Cases with non-excellent response outcome (n=153)** | **Univariable** | | **Multivariable** | | |
| --- | --- | --- | --- | --- | --- | --- | --- | --- |
|  |  |  |  | **OR (95% CI)** | **P-value** | **Adjusted  OR (95% CI)** | | **P-value** |
| **sTg/TSH ratio subgroup (median 0.2)** | | | | | |  |  | |
| Subgroup 1 | 62  (100%) | 27  (43.5%) | 35  (56.5%) | Ref. |  |  |  | |
| Subgroup 2 | 64  (100%) | 9  (14%) | 55  (86%) | 4.71  (1.98,11.2) | <0.001 |  |  | |
| Subgroup 4 | 7  (100%) | 0  (0%) | 7  (100%) | NA |  |  |  | |
| Subgroup 6 | 4  (100%) | 0  (0%) | 4  (100%) | NA |  |  |  | |
| Subgroup 7 | 90  (100%) | 38  (42%) | 52  (58%) | 1.06  (0.55,2.03) | 0.871 |  |  | |
| **sTg/TSH ratio subgroup** | | | | | |  |  | |
| Subgroup  1, 3, 5, 7 | 152  (100%) | 65  (43%) | 87  (57%) | Ref. |  |  |  | |
| Subgroup  2, 4, 6 | 75  (100%) | 9  (12%) | 66  (88%) | 5.48 (2.54,11.8) | <0.001 |  |  | |
| **Subgroup 7: TSH >100 µIU/mL and sTg has a reportable value** | | | | | | | | |
| Tg <9.4 | 52  (100%) | 30  (58%) | 22  (42%) | Ref. |  |  |  | |
| Tg ≥9.4 | 38  (100%) | 8  (21%) | 30  (79%) | 5.11  (1.97,13.28) | 0.001 |  |  | |
