# Supplementary material for: The effects of stimulated thyroglobulin/ thyroid-stimulating hormone ratio and N1b status on the efficacy of the first I-131 treatment in patients with differentiated thyroid cancer
Source: PLoS One. 2026 Aug 17;21(8):e0349850. doi: 10.1371/journal.pone.0349850 (PMC13480613; doi:10.1371/journal.pone.0349850)
Supplement: Supplementary file 3 [file pone.0349850.s005.docx]

**Authors’ revision in response to reviewers’ comments**

Dear Reviewers and Editor-in-Chief of Plos One

I have received comments from the reviewers and would like to extend my thanks and genuine appreciation to the reviewers and editorial team who took their valuable time to read and provide comprehensive comments to help improve this manuscript. I have addressed the reviewers’ comments as follows. Corrections are highlighted in yellow in the revised manuscript.

| **Reviewer’s comments** | **Authors’ response and action** |
| --- | --- |
| **Reviewer #1** |  |
| 1. 1. 2. It would be helpful to provide more information about the radioactive iodine treatment protocol at your center.   Is there standardized treatment at your center? This information is important for the reader to know if changes in treatment regimen may have affected the results. | **Response**:  Thank you for the suggestion. Yes, there is an institutional protocol.  **Action**: Added details of the institutional protocol in the methods section.  **Initial MS**: Generally, at our center radioactive iodine dosage in low risk and intermediate-high risk patients was 30 mCi and 150-200 mCi, respectively; however, in low-risk patients with T3-T4 tumor, the attending nuclear medicine physician might be prescribed dosage in the 100-150 mCi range.  **Revised MS**: Radioactive iodine treatment follows an institutional protocol. The dosage radioactive iodine corresponds with ATA risk of recurrence category and tumor staging. A dosage of 30 mCi is prescribed for patients with low-risk of recurrence and T1 or T2 disease. A dosage of 100 mCi is prescribed for low-risk of recurrence and T3 disease. A dosage of 150 mCi is prescribe for patients with intermediate and high-risk patients as well as those with distant lung metastases and those with T4 disease. A dosage of 200 mCi is reserved for patients with bone metastases.  **Location of revision:** Lines 106-112 |
| 2.  The use of a significant level of p < 0.2 in univariate regression may be confusing to some readers. Typically, a p-value < 0.05 is used in both steps. If there is a specific reason for choosing a p-value level of < 0.2 in this step, it is suggested that a brief explanation be provided. This will help clarify your reasoning for this choice. | **Response**:  From consultation with statistician and some information regarding the multivariate logistic regression analysis (<https://pmc.ncbi.nlm.nih.gov/articles/PMC7877407/>). We decided to set the significant p-value in the univariate analysis in more liberal fashion (p-value <0.2) to reduce the possibility of insignificant variable in univariate logistic regression analysis but significant in multivariable analysis.  **Action**: Added additional explanation in the methods section.  **Initial MS**: Logistic regression analysis was used to find significant variables. Univariable logistic regression was performed initially to screen variables, using a significance level of p-value <0.2 at this stage.  **Revised MS**: Logistic regression was used to determine predictive factors of radioactive iodine treatment outcome. First, univariable logistic regression was performed on all predictors to screen for candidate variables. At this stage, predictors with a p-value of <0.2 was considered as a potential candidate and proceeded to be analyzed in the multivariable analysis. This liberal p-value threshold was chosen to avoid excluding potential important predictors which could turn out to be significant in the multivariable model.  **Location of revision:** Lines 130-135 |
| 3.   The section on dividing the data into subgroups is well explained but needs further clarification. Why were these specific combinations chosen for the subgroup division?   4.   In cases where Tg values < 0.04 or >500 ng/mL or TSH > 100 µIU/mL are reported, a clear explanation of how these values were handled in the analyses appears to be provided.  However, further clarification of how these missing or incorrect data are handled (such as omitting these data or using alternative methods for analysis) would be useful.  Especially in subgroups where these specific values exist, mention whether incomplete data were removed from the analyses or how you dealt with these values. | **Response**: Revised as recommended.  - Combination of subgroups 1, 3, 5 and 7 and combination of subgroups 2, 4 and 6 is based on the rationale that sTg/TSH ratio might tend to be lower and higher in each combination, respectively.  - The patient without key laboratory values data (TSH, Tg, TgAb) is excluded.  **Action**: Add additional explanation in the methods section  **Initial MS**: not mentioned  **Revised MS**:  “Patients were excluded if no reported sTg, TSH and TgAb”  **Location of revision:** Line 91  “There were combinations of these subgroups in the analysis; combination of subgroups 1, 3, 5 and 7 and combination of subgroups 2, 4 and 6 based on the rationale that sTg/TSH ratio might tend to be lower and higher in each combination, respectively.”  **Location of revision:** Lines 150-153 |
| 5.   Further explanation on how lymph node metastasis was assessed in the study (was advanced imaging used?) and how metastasis was detected at an early stage. | **Response**: Revised as recommended.  **Action**: Add additional explanation in the methods section  **Initial MS**: not mentioned  **Revised MS**: “Determination of nodal and distant metastasis status is based on tissue diagnosis from surgery or radioiodine-avid lesion from the post-treatment WBS.”  **Location of revision:** Lines 103-104 |
| 6.   However, it is suggested that more explanation be provided in the ROC analysis about the sensitivity and specificity at these thresholds. | **Response**: Revised as recommended.  **Action**: Added additional details in table 2, and added discussion regarding the ROC analysis and effects of cutoff values in the discussion section.  **Initial MS**: -  **Revised MS**:    **Location of revision:** Line 198  “The ability of sTg and the sTg/TSH ratio to differentiate between patients with excellent and non-excellent response outcomes, as shown by the ROC analysis, was found to be rather similar based on the shape of the graph, AUC values and also the cut-off values from the median and Youden index were rather close, at 9.4 and 10.1 ng/dL, respectively. However, it is notable that this minimal shift in the cutoff point causes the sensitivities and specificities to be “flipped”, with the cutoff of 9.4 ng/mL having a sensitivity of 65% and a specificity of 77%, while moving the cutoff to 10.1 ng/dL results in a higher sensitivity of 81% and a lower specificity of 67%. This can be explained by “datapoint clumping” with many of the patients with excellent response, and non-excellent response having the sTg around these threshold boundaries. That being said, in our opinion, a cutoff of 10.1 ng/dL based on the Youden index would be more appropriate as it gives a higher sensitivity and an acceptable specificity. This cutoff is also similar to the findings of a large study by Webb et al. in 2012 [24]..”  **Location of revision:** Lines 263-270 |
| 7.   In the analysis section, age is discussed in detail, but it seems that further explanation of why age ≤45 years is not effective as an independent factor in some studies would be valuable. Is this due to a lack of evidence in small samples, or are different analysis methods in different studies causing different results? | **Response**: Revised as recommended.  **Action**: Add additional explanation  **Initial MS**: In Discussion section    **Revised MS**: “For age, although it was generally believed that increasing age was associated with worse disease progression, some studies had found that age was not an independent risk factor for a poor treatment outcome. Thamnirat et al. in 2015 [22] in 256 patients found that age ≤45 years was not associated with disease-free status in multivariate analysis. Additionally, a study by Ganly et al. in 2015 [23] involving 3,664 thyroid cancer patients found that when age was considered as a continuous variable affecting disease-specific survival, the adjusted hazard ratio (aHR) was 1.076 (95%CI:1.06–1.1, p-value<0.001). The confidence interval was close to 1, the null hypothesis, thus studies with smaller populations, such as this study and Thamnirat et al., might not have sufficient power enough to find age as a significant factor. ”  **Location of revision**: Lines 246-252 |
| **Reviewer #2** |  |
| 1.  The manuscript relies on the ATA 2015 guidelines, suggesting that risk stratification and treatment response assessment were based on an outdated framework. Since then, significant updates have been introduced, including the 2022 update and the more comprehensive 2025 ATA guidelines. Continued reliance on ATA 2015 may limit alignment with current standards of care, particularly regarding recurrence risk stratification, RAI dosing strategies, and management of patients with N1b disease. The authors should either update their framework in accordance with ATA 2025 or explicitly acknowledge these updates in the Discussion and justify the use of the earlier version. | **Response**: Revised as recommended.  **Action**: Patients included in the study up to 31 December 2023 have been managed according to ATA 2015 guideline. However, there is a new ATA guideline published in 2025. Thus, we add this information to the Discussion section as recommended.  **Initial MS**: not mentioned  **Revised MS**:” There were several limitations of this study. First, this study has been conducted before the publication of new ATA guideline for differentiated thyroid cancer in 2025. Patients treated at our center were managed based on the now superseded ATA 2015 guidelines, thus there might be some difference in risk stratification and management of the patient.”  **Location of revision:** Lines 277-280 |
| 2.  The study’s retrospective, single-center design inherently limits control of confounding variables and reduces external validity. As the research was conducted in a single institution in Thailand, the generalizability of the findings to other populations and healthcare systems may be restricted. | **Response**: Revised as recommended.  **Action**: Added details to emphasize this limitation in Discussion section  **Initial MS**: -  **Revised MS**: “Second, due to retrospective single-center design of the study, this might lead to incomplete data for some variables, limitation in control of confounders and the data should be considered before applying to other population.”  **Location of revision:** Lines 280-282 |
| 3.  The change in laboratory assay methodology (from RIA to ECLIA) and the exclusion of earlier data potentially introduced selection bias and reduced the effective sample size. While the timeline, laboratory methods, and exclusion criteria are clearly described, inclusion of certain operational details (e.g., exact dates of data access approval) appears unnecessary and may detract from the methodological clarity of the manuscript. | **Response**: Revised as recommended  **Action**:  - Add some additional details regarding the difference between previous and current methods of laboratory technique which could not be compared and have been excluded.  - Delete the unnecessary information. It may be worth noting that inclusion of such a detailed description of dates and approval timeline were in response to the editorial comments at the time of initial submission.  **Initial MS**: In methods section    **Revised MS**: “Initial ethics approval was granted on 13 June 2023 which allowed access to retrospective data from 1 January 2018 to 31 December 2022. After preliminary analysis of data in this period, it was found that data older than 31 December 2019 were not appropriate for analysis since the laboratory technique used at the time was radioimmunoassay which was later replaced by chemiluminescence immunoassay. The reference range of these two methods was different and could not be directly compared to each other. Therefore, an amendment request was made to the ethics committee to change the data range from 1 January 2020 to 31 December 2023. The amendment was approved on 7 April 2024.”  **Location of revision:** Lines 78-85 |
| 4.  the limited sample size within certain subgroups and the presence of multicollinearity among sTg, the sTg/TSH ratio, and other covariates likely contributed to the loss of statistical significance of key variables (notably the sTg/TSH ratio and N1b status) in multivariable regression analysis. Despite this, the Discussion emphasizes the potential usefulness of the sTg/TSH ratio, which is not supported by the adjusted model. This creates inconsistency between statistical findings and interpretation. | **Response**: Revise as recommended.  **Action**: We adjust some parts in the discussion to more accurately reflect the results.  **Initial MS**: In Discussion section  (1)    (2)    **Revised MS**: In Discussion section  (1) Omit this part of discussion to reduce inconsistency.  (2) Added “As for the sTg/TSH ratio, a similar phenomenon was found when analyzing the cutoff values. Moreover, the cutoff of 0.23 based on the Youden index yielded a sensitivity of 86% at the expense of a lower specificity of 60% compared with sTg. Therefore, the sTg/TSH ratio does not reveal superiority to sTg based on these current findings.”  **Location of revision:** Lines 273-276 |
| 5.  the follow-up period of 6–18 months primarily reflects short-term treatment response rather than long-term prognosis. Given the typically indolent course of DTC, longer follow-up would be necessary to assess recurrence and durable outcomes. | **Response**: Revise as recommended.  **Action**: Add additional explanation  **Initial MS**: not mentioned  **Revised MS**: “Lastly, due to the rather indolent disease course of thyroid cancer, limited follow-up time might not truly reflect the long-term outcome of the patients, thus longer follow-up time was encouraged in further studies.”  **Location of revision:** Lines 288-290 |
| 6.  Important lymph node characteristics (such as size and number of metastatic nodes), which may influence prognosis, were not fully analyzed. | **Response**: Revise as recommended.  **Action**: Add additional explanation  **Initial MS**: Not mention  **Revised MS**: “For other lymph node characteristics such as numbers, size, and extranodal extension, these factors were analyzed in the univariate analysis, as shown in S2 Table. None of the factors showed significance in predicting treatment outcome. Due to the fact that some patients were referred from other hospitals, completed details of lymph node characteristics could not be obtained for some cases.”  **Location of revision:** Lines 255-259 |
| 7.  The analysis of RAI dosage may be affected by confounding by indication. Higher RAI doses (150–200 mCi) were likely administered to patients with more advanced disease or higher ATA risk categories. Therefore, the observed association between higher RAI dose and poorer treatment response may reflect baseline disease severity rather than an independent causal effect of the administered dose. The manuscript does not clearly demonstrate adequate statistical adjustment for this potential bias. Stratified analysis within risk categories or the use of propensity score methods would strengthen internal validity. | **Response**: Revise as recommended.  **Action**: Adjust the discussion to address this potential bias.  **Initial MS**: In the Discussion section    **Revised MS**: “For the radioactive iodine dosage, patients receiving higher radioiodine dosage often had a poorer prognosis; this is likely due to their baseline disease severity rather than the treatment itself.”  **Location of revision:** Lines 240-242 |
| 8.  There appears to be a discrepancy between the primary objective and the final conclusions. The study aimed to evaluate the impact of the sTg/TSH ratio and N1b status on treatment response; however, neither variable remained significant in multivariable analysis. Ultimately, only sTg was identified as an independent predictor — a finding already well established in prior literature. Consequently, the degree of novelty is limited. | **Response**: Revise as recommended.  **Action**: We adjust the conclusion section to be more focused on the primary objective.  **Initial MS**: In the Conclusion section    **Revised MS**: “The sTg/TSH ratio and the presence of lateral cervical lymph node metastasis (N1b) were not independent risk factors for a non-excellent response in this study, which could be due to a relatively small sample size in the context of the presence of multicollinearity with other well-established predictors such as sTg.”  **Location of revision:** Lines 293-296 |

Once again, thank you for considering this manuscript, and I hope this revision adequately addressed the issues raised by the reviewers. If there are any other issues that need to be addressed, I would be glad to do so.

Sincerely,

Corresponding author.
