# Supplementary material for: The effects of stimulated thyroglobulin/ thyroid-stimulating hormone ratio and N1b status on the efficacy of the first I-131 treatment in patients with differentiated thyroid cancer
Source: PLoS One. 2026 Aug 17;21(8):e0349850. doi: 10.1371/journal.pone.0349850 (PMC13480613; doi:10.1371/journal.pone.0349850)
Supplement: Supplementary file 4 [file pone.0349850.s006.docx]

**Authors’ revision in response to reviewers’ comments**

Dear Reviewers and Editor-in-Chief of Plos One

I have received comments from the reviewers and would like to extend my thanks and genuine appreciation to the reviewers and editorial team who took their valuable time to read and provide comprehensive comments to help improve this manuscript. I have addressed the reviewers’ comments as follows. Corrections are highlighted in yellow in the revised manuscript.

| **Reviewer’s comments** | **Authors’ response and action** |
| --- | --- |
| **Reviewer #2** | No comments |
| **Reviewer #3** |  |
| #Add a formal sample size/power justification: The manuscript does not describe how the sample size was determined or whether the study was adequately powered to detect meaningful effects, which limits confidence in negative findings. | **Response**: Revised as recommended.  **Action**:  1. Added a dedicated “Sample size considerations” subsection in the Methods section.  2. Power calculation was performed.  **Initial MS**: The population included in the analysis was 227 subjects.  **Revised MS**:  **[Methods]**  **Sample size considerations**  All patient records that are available in the research data collection period which met the aforementioned inclusion and exclusion criteria were included in the analysis. To determine the adequacy of the final sample size, power calculation was done using the R package, WebPower. The statistical power was determined based on the difference of the prevalence of non-excellent response in cases with a positive versus negative predictor of interest, and a significance level of 0.05.  **[Results]**  The population included in the analysis was 227 subjects, among which 153 (67.4%) had non-excellent response outcome. Among patients with N1b node status the non-excellent outcome was 82%, as compared with 60% in those without N1b lymph nodes. This resulted in a statistical power of approximately 89%.  **Location of revision:** Lines 165-171, and 172-175 |
| #Cite the statistical software: Specify the statistical software (e.g., SAS, R, SPSS) used for the analysis to improve reproducibility and transparency. | **Response**:  Revised as recommended.  **Action**: Add statistical software in the method section.  **Initial MS**: -  **Revised MS**:  Descriptive statistics and logistic regression were performed using STATA version 18 (StataCorp LLC, TX, USA). ROC analysis, which was analyzed using R Statistical Software (version 4.2.1; R Core Team), RStudio version 2023.12.1+402 "Ocean Storm" (RStudio, Boston, MA) and the R package, pROC. Power calculation was done using the R package, WebPower.  **Location of revision:** Lines 161-164 |
| #Additional details on model evaluation, such as assessment of multicollinearity (e.g., variance inflation factors) and goodness-of-fit, should be provided to support the validity of the regression results. | **Response**:  Revised as recommended.  **Action**: Add parameters regarding the logistic regression analysis in the result section  **Initial MS**: -  **Revised MS**:  Multivariable logistic regression analysis was performed on 227 participants. Model performance was evaluated using the Akaike Information Criterion (AIC = 223.6) and Hosmer-Lemeshow goodness-of-fit test (χ²(8) = 8.62, p = 0.376), confirming adequate model fit.  **Location of revision:** Lines 187-189 |
| #The manuscript should explicitly state how missing data were handled (e.g., exclusion vs imputation), as this can influence both estimates and generalizability. | **Response**:  1. If the missing data was important primary investigated factors such as sTg, TSH and TgAb, no SPECT/CT of the neck with the post-treatment whole body scan (WBS), unclear staging or risk of recurrence, missing data of treatment response evaluation), The patient would be excluded.  2. Regarding missing of other secondary variables of interest, overall missing data rare since almost all cases had complete medical records regarding the important clinical factors. Referring to table 1 (logistic regression results), the only variable with missing data is the ETE status, due to a lack of specific reporting the pathology results. For this variable, we labeled the cases where ETE status was not specifically mentioned in the pathology report as ‘unknown’ when performing logistic regression. The results have been added to table 1.  **Initial MS**: In the Methods section, exclusion of cases with missing outcome variable or main predictor variable has already been mentioned in lines 91-97.  **Revised MS**: [Added analysis of results of cases where the ETE status is ‘unknown’]    **Location of revision:** Table 1 |
| #Provide events-per-variable justification: Report the number of outcome events and comment on the events-per-variable ratio to ensure the multivariable model is not overfit and results are stable. | **Response**: A total of 5 variables went into the multivariable logistic regression, using the Peduzzi's Rule of 10 events per variable, at least 50 events were needed for reliable results. In our study, there are a total of 153 events, therefore adequate for the analysis.  **Initial MS**: -  **Revised MS**:  [Results]  Since a total of 5 variables were included in the multivariable logistic regression analysis, at least 50 events were needed according to the 10-events per variable rule. In this study, there are a total of 153 events, which is adequate for a reliable regression model.  **Location of revision:** 192-194 |
| #Table 1: For the cervical node metastasis location and sTg/TSH ratio subgroups, first report an overall p-value testing for differences across all groups, followed by appropriate stepdown (post hoc) comparisons to identify which specific group pairs differ. | **Response**: We thank the reviewer for this comment. This aspect was omitted as the relevant parameters did not reach statistical significance on logistic regression analysis which was primary analysis. |
| #Subgroup analysis rationale remains weak: The explanation for subgroup construction is still ad hoc and lacks a clear statistical or prespecified justification.  And  #Handling of extreme/censored values is unclear: Although some exclusions are described, the analytical approach for values below/above assay limits is not fully specified and may introduce bias. | **Response**: Revised as recommended.  **Action**: We added a more detailed explanation of this concern in the Methods section.  **Initial MS**:  In cases where a variable was reported as exceeding the upper limit or falling below the lower limit of reporting in laboratory tests, such as sTg <0.04, >500 ng/mL or TSH >100 µIU/mL, it was impossible to calculate the true numerical value of sTg/TSH ratio. The analysis would be performed in two ways; by excluding the data from the population group whose variable values exceeding the upper limit or falling below the lower limit i.e. sTg <0.04, >500 ng/mL or TSH >100 µIU/mL and by dividing into various subgroups. There were total seven subgroups with different combinations of sTg/TSH ratio and TSH as follows: subgroup 1, sTg/TSH ratio < mean or median; subgroup 2, sTg/TSH ratio ≥ mean or median; subgroup 3, TSH having a reportable value and sTg <0.04 ng/mL; subgroup 4, TSH having a reportable value and sTg >500 ng/mL; subgroup 5, TSH >100 µIU/mL and sTg <0.04 ng/mL; subgroup 6, TSH >100 µIU/mL and sTg >500 ng/mL; and subgroup 7, TSH >100 µIU/mL and sTg having a reportable value. There were combinations of these subgroups in the analysis; combination of subgroups 1, 3, 5 and 7 and combination of subgroups 2, 4 and 6 based on the rationale that sTg/TSH ratio might tend to be lower and higher in each combination, respectively.  **Revised MS**:  In cases where extreme values were present, for example variables reported as exceeding the upper limit or falling below the lower limit in laboratory tests, such as sTg <0.04 ng/mL, sTg >500 ng/mL, or TSH >100 µIU/mL, it was impossible to calculate the true numerical value of the sTg/TSH ratio. This was not uncommon in real-world clinical practice. The analysis was therefore performed in two ways: first, by excluding data from patients whose variable values exceeded the upper limit or fell below the lower limit of detection, i.e., sTg <0.04 ng/mL, sTg >500 ng/mL, or TSH >100 µIU/mL; and second, by dividing the population into subgroups to cover all potential combinations of sTg and TSH results.  A total of seven subgroups were defined based on different combinations of sTg, TSH, and the sTg/TSH ratio, as follows. Subgroup 1 comprised patients with both sTg and TSH within reportable ranges and sTg/TSH ratio < mean or median. Subgroup 2 comprised patients with both sTg and TSH within reportable ranges and sTg/TSH ratio ≥ mean or median. Subgroup 3 included patients with TSH within a reportable range and sTg <0.04 ng/mL. Subgroup 4 included patients with TSH within a reportable range and sTg >500 ng/mL. Subgroup 5 included patients with TSH >100 µIU/mL and sTg <0.04 ng/mL. Subgroup 6 included patients with TSH >100 µIU/mL and sTg >500 ng/mL. Subgroup 7 included patients with TSH >100 µIU/mL and sTg within a reportable range.  Each subgroup was analyzed independently using logistic regression. The subgroups were also further combined into two broader groups based on clinical perspective: a potential low Tg and low sTg/TSH ratio group, comprising Subgroups 1, 3, 5, and 7, and a potential high Tg and high sTg/TSH ratio group, comprising Subgroups 2, 4, and 6. These two broader groups were also analyzed by logistic regression.  **Location of revision:** Line 140-160 |
| #ROC analysis lacks formal comparison: The added discussion is descriptive; no statistical comparison of AUCs or model performance is provided | **Response**: We used DeLong’s test to compare the AUCs of the ROC curves which yielded a p-value of 0.729 indicating no significant difference.  **Initial MS**: -  **Revised MS**:  DeLong’s test yielded a p-value of 0.729 which indicates no significant difference between the area under the curves of the two ROCs.  **Location of revision:** Line 203-204 |
| #Confounding by indication not addressed analytically: The issue is acknowledged, but no statistical adjustment (e.g., stratification or propensity methods) was performed.  And  #Limited novelty only reframed: | **Response**: We carefully considered the issues raised in this comment and we believe that these issues do not apply to this specific study, because they are biases specific to observational studies of effect of an exposure (or treatment) on an outcome. “Confounding by indication” is when a confounding variable affects both the exposure and the outcome of interest, cf. <https://catalogofbias.org/biases/confounding-by-indication/>. Our study simply studies the prognostic implications of clinical factors on the successfulness of I-131 treatment prescribed according to standard medical care, and therefore is not affected by this bias. Propensity methods such as propensity score matching are used to reduce effects of confounding factors in observational studies of treatment effects by adjusting for the probability of treatment assignment. It’s a method used to ‘emulate’ a randomized controlled trial using observational data, therefore this method is not relevant to this current study. Regarding dealing with confounders, the multivariable regression which was already done should be sufficient. |

Once again, thank you for considering this manuscript, and I hope this revision adequately addressed the issues raised by the reviewers.

Sincerely,

Corresponding author.
