# Supplementary material for: The effects of stimulated thyroglobulin/ thyroid-stimulating hormone ratio and N1b status on the efficacy of the first I-131 treatment in patients with differentiated thyroid cancer
Source: PLoS One. 2026 Aug 17;21(8):e0349850. doi: 10.1371/journal.pone.0349850 (PMC13480613; doi:10.1371/journal.pone.0349850)
Supplement: Peer review history

# Peer review: Decision Letter 0

**DOI:** 10.1371/journal.pone.0349850.r001

**Authors:** Afagh Hassanzadeh Rad

16 Feb 2026

Dear Dr. Theerakulpisut,

Please submit your revised manuscript by Apr 02 2026 11:59PM. If you will need more time than this to complete your revisions, please reply to this message or contact the journal office at plosone@plos.org. When you're ready to submit your revision, log on to https://www.editorialmanager.com/pone/ and select the 'Submissions Needing Revision' folder to locate your manuscript file.

- A letter that responds to each point raised by the academic editor and reviewer(s). You should upload this letter as a separate file labeled 'Response to Reviewers'.
- A marked-up copy of your manuscript that highlights changes made to the original version. You should upload this as a separate file labeled 'Revised Manuscript with Track Changes'.
- An unmarked version of your revised paper without tracked changes. You should upload this as a separate file labeled 'Manuscript'.

If you would like to make changes to your financial disclosure, please include your updated statement in your cover letter. Guidelines for resubmitting your figure files are available below the reviewer comments at the end of this letter.

If applicable, we recommend that you deposit your laboratory protocols in protocols.io to enhance the reproducibility of your results. Protocols.io assigns your protocol its own identifier (DOI) so that it can be cited independently in the future. For instructions see: https://journals.plos.org/plosone/s/submission-guidelines#loc-laboratory-protocols. Additionally, PLOS ONE offers an option for publishing peer-reviewed Lab Protocol articles, which describe protocols hosted on protocols.io. Read more information on sharing protocols at https://plos.org/protocols?utm_medium=editorial-email&utm_source=authorletters&utm_campaign=protocols.

We look forward to receiving your revised manuscript.

Kind regards,

Afagh Hassanzadeh Rad

Academic Editor

PLOS One

Journal Requirements:

When submitting your revision, we need you to address these additional requirements.

1. Please ensure that your manuscript meets PLOS ONE's style requirements, including those for file naming. The PLOS ONE style templates can be found at

https://journals.plos.org/plosone/s/file?id=wjVg/PLOSOne_formatting_sample_main_body.pdf and https://journals.plos.org/plosone/s/file?id=ba62/PLOSOne_formatting_sample_title_authors_affiliations.pdf

2. Please include a caption for figure 3.

3. We note that there is identifying data in the Supporting Information file <S3_dataset.xlsx>. Due to the inclusion of these potentially identifying data, we have removed this file from your file inventory. Prior to sharing human research participant data, authors should consult with an ethics committee to ensure data are shared in accordance with participant consent and all applicable local laws.

Data sharing should never compromise participant privacy. It is therefore not appropriate to publicly share personally identifiable data on human research participants. The following are examples of data that should not be shared:

-Name, initials, physical address

-Ages more specific than whole numbers

-Internet protocol (IP) address

-Specific dates (birth dates, death dates, examination dates, etc.)

-Contact information such as phone number or email address

-Location data

-ID numbers that seem specific (long numbers, include initials, titled “Hospital ID”) rather than random (small numbers in numerical order)

Data that are not directly identifying may also be inappropriate to share, as in combination they can become identifying. For example, data collected from a small group of participants, vulnerable populations, or private groups should not be shared if they involve indirect identifiers (such as sex, ethnicity, location, etc.) that may risk the identification of study participants.

Additional guidance on preparing raw data for publication can be found in our Data Policy (https://journals.plos.org/plosone/s/data-availability#loc-human-research-participant-data-and-other-sensitive-data) and in the following article: http://www.bmj.com/content/340/bmj.c181.long.

Please remove or anonymize all personal information (<specific identifying information in file to be removed>), ensure that the data shared are in accordance with participant consent, and re-upload a fully anonymized data set. Please note that spreadsheet columns with personal information must be removed and not hidden as all hidden columns will appear in the published file.

4. If the reviewer comments include a recommendation to cite specific previously published works, please review and evaluate these publications to determine whether they are relevant and should be cited. There is no requirement to cite these works unless the editor has indicated otherwise.

[Note: HTML markup is below. Please do not edit.]

Reviewers' comments:

Reviewer's Responses to Questions

**Comments to the Author**

1. Is the manuscript technically sound, and do the data support the conclusions?

Reviewer #1: Partly

Reviewer #2: No

**********

2. Has the statistical analysis been performed appropriately and rigorously?

Reviewer #1: I Don't Know

Reviewer #2: No

**********

3. Have the authors made all data underlying the findings in their manuscript fully available?

The PLOS Data policy

Reviewer #1: Yes

Reviewer #2: No

**********

4. Is the manuscript presented in an intelligible fashion and written in standard English?

Reviewer #1: Yes

Reviewer #2: Yes

**********

Reviewer #1: It would be helpful to provide more information about the radioactive iodine treatment protocol at your center. Is there a standardized treatment at your center? This information is important for the reader to know if changes in treatment regimen may have affected the results.

The use of a significance level of p < 0.2 in univariate regression may be confusing to some readers. Typically, a p-value < 0.05 is used in both steps. If there is a specific reason for choosing a p-value level of < 0.2 in this step, it is suggested that a brief explanation be provided. This will help clarify your reasoning for this choice.

The section on dividing the data into subgroups is well explained, but needs further clarification. Why were these specific combinations chosen for the subgroup division?

These can explain to readers why this particular approach was chosen and how it might affect the results.

In cases where Tg values < 0.04 or >500 ng/mL or TSH > 100 µIU/mL are reported, a clear explanation of how these values were handled in the analyses appears to be provided. However, further clarification of how these missing or incorrect data are handled (such as omitting these data or using alternative methods for analysis) would be useful.

Especially in subgroups where these specific values exist, mention whether incomplete data were removed from the analyses or how you dealt with these values.

Further explanation on how lymph node metastasis was assessed in the study (was advanced imaging used?) and how metastasis was detected at an early stage. However, it is suggested that more explanation be provided in the ROC analysis about the sensitivity and specificity at these thresholds.

In the analysis section, age is discussed in detail, but it seems that further explanation of why age ≤45 years is not effective as an independent factor in some studies would be valuable.Is this due to a lack of evidence in small samples, or are different analysis methods in different studies causing different results?

Ultimately, your article is very valid and useful, and by making these corrections, its scientific credibility will increase

Reviewer #2: Dear Editor

Thank you for the opportunity to review this manuscript.

My comments are as follows:

This manuscript addresses an important and clinically relevant topic, namely the prediction of response to radioactive iodine (RAI) therapy in patients with differentiated thyroid cancer (DTC). The finding regarding sTg ≥ 9.4 ng/mL is noteworthy, and the use of internationally recognized sources such as GLOBOCAN and ATA guidelines reflects an appropriate scientific foundation. However, the study has several important limitations that require substantial revision prior to consideration for publication.

First, the manuscript relies on the ATA 2015 guidelines, suggesting that risk stratification and treatment response assessment were based on an outdated framework. Since then, significant updates have been introduced, including the 2022 update and the more comprehensive 2025 ATA guidelines. Continued reliance on ATA 2015 may limit alignment with current standards of care, particularly regarding recurrence risk stratification, RAI dosing strategies, and management of patients with N1b disease. The authors should either update their framework in accordance with ATA 2025 or explicitly acknowledge these updates in the Discussion and justify the use of the earlier version.

Second, the study’s retrospective, single-center design inherently limits control of confounding variables and reduces external validity. As the research was conducted in a single institution in Thailand, the generalizability of the findings to other populations and healthcare systems may be restricted.

Third, the change in laboratory assay methodology (from RIA to ECLIA) and the exclusion of earlier data potentially introduced selection bias and reduced the effective sample size. While the timeline, laboratory methods, and exclusion criteria are clearly described, inclusion of certain operational details (e.g., exact dates of data access approval) appears unnecessary and may detract from the methodological clarity of the manuscript.

Fourth, the limited sample size within certain subgroups and the presence of multicollinearity among sTg, the sTg/TSH ratio, and other covariates likely contributed to the loss of statistical significance of key variables (notably the sTg/TSH ratio and N1b status) in multivariable regression analysis. Despite this, the Discussion emphasizes the potential usefulness of the sTg/TSH ratio, which is not supported by the adjusted model. This creates inconsistency between statistical findings and interpretation.

Fifth, the follow-up period of 6–18 months primarily reflects short-term treatment response rather than long-term prognosis. Given the typically indolent course of DTC, longer follow-up would be necessary to assess recurrence and durable outcomes.

Sixth, important lymph node characteristics (such as size and number of metastatic nodes), which may influence prognosis, were not fully analyzed.

Seventh, the analysis of RAI dosage may be affected by confounding by indication. Higher RAI doses (150–200 mCi) were likely administered to patients with more advanced disease or higher ATA risk categories. Therefore, the observed association between higher RAI dose and poorer treatment response may reflect baseline disease severity rather than an independent causal effect of the administered dose. The manuscript does not clearly demonstrate adequate statistical adjustment for this potential bias. Stratified analyses within risk categories or the use of propensity score methods would strengthen internal validity.

Finally, there appears to be a discrepancy between the primary objective and the final conclusions. The study aimed to evaluate the impact of the sTg/TSH ratio and N1b status on treatment response; however, neither variable remained significant in multivariable analysis. Ultimately, only sTg was identified as an independent predictor — a finding already well established in prior literature. Consequently, the degree of novelty is limited.

In light of these considerations, the manuscript requires major revision, including updating the guideline framework, refining the statistical modeling, simplifying subgroup analyses, and providing a clearer and more balanced discussion of limitations and clinical implications, before it can be considered for publication in a reputable journal.

Sincerely

**********

what does this mean?). If published, this will include your full peer review and any attached files.

If you choose “no”, your identity will remain anonymous but your review may still be made public.

**Do you want your identity to be public for this peer review?**  For information about this choice, including consent withdrawal, please see our Privacy Policy

Reviewer #1: **Yes:** Sara Nikpour

Reviewer #2: No

**********

[NOTE: If reviewer comments were submitted as an attachment file, they will be attached to this email and accessible via the submission site. Please log into your account, locate the manuscript record, and check for the action link "View Attachments". If this link does not appear, there are no attachment files.]

To ensure your figures meet our technical requirements, please review our figure guidelines: https://journals.plos.org/plosone/s/figures

You may also use PLOS’s free figure tool, NAAS, to help you prepare publication quality figures: https://journals.plos.org/plosone/s/figures#loc-tools-for-figure-preparation.

NAAS will assess whether your figures meet our technical requirements by comparing each figure against our figure specifications.

# Author comment: Author response to Decision Letter 1

**DOI:** 10.1371/journal.pone.0349850.r002

26 Feb 2026

Response to reviewers can be found in the attached "Response to reviewers.docx" file.

Attachment Submitted filename: Response to reviewers.docx

# Peer review: Decision Letter 1

**DOI:** 10.1371/journal.pone.0349850.r003

**Authors:** Alejandro Torrado Pacheco

11 Jun 2026

Dear Dr. Theerakulpisut,

Thank you for submitting your manuscript to PLOS ONE. After careful consideration, we feel that it has merit but does not fully meet PLOS ONE’s publication criteria as it currently stands. Therefore, we invite you to submit a revised version of the manuscript that addresses the points raised during the review process.

The manuscript has been evaluated by two reviewers, and their comments are available below.

Could you please revise the manuscript to carefully address the concerns raised?

Please submit your revised manuscript by Jul 26 2026 11:59PM. If you will need more time than this to complete your revisions, please reply to this message or contact the journal office at plosone@plos.org. When you're ready to submit your revision, log on to https://www.editorialmanager.com/pone/ and select the 'Submissions Needing Revision' folder to locate your manuscript file.

- A letter that responds to each point raised by the academic editor and reviewer(s). You should upload this letter as a separate file labeled 'Response to Reviewers'.
- A marked-up copy of your manuscript that highlights changes made to the original version. You should upload this as a separate file labeled 'Revised Manuscript with Track Changes'.
- An unmarked version of your revised paper without tracked changes. You should upload this as a separate file labeled 'Manuscript'.

If you would like to make changes to your financial disclosure, please include your updated statement in your cover letter. Guidelines for resubmitting your figure files are available below the reviewer comments at the end of this letter.

If applicable, we recommend that you deposit your laboratory protocols in protocols.io to enhance the reproducibility of your results. Protocols.io assigns your protocol its own identifier (DOI) so that it can be cited independently in the future. For instructions see: https://journals.plos.org/plosone/s/submission-guidelines#loc-laboratory-protocols. Additionally, PLOS ONE offers an option for publishing peer-reviewed Lab Protocol articles, which describe protocols hosted on protocols.io. Read more information on sharing protocols at https://plos.org/protocols?utm_medium=editorial-email&utm_source=authorletters&utm_campaign=protocols.

As the corresponding author, your ORCID iD is verified in the submission system and will appear in the published article. PLOS supports the use of ORCID, and we encourage all coauthors to register for an ORCID iD and use it as well. Please encourage your coauthors to verify their ORCID iD within the submission system before final acceptance, as unverified ORCID iDs will not appear in the published article. *Only*  the individual author can complete the verification step; PLOS staff *cannot*  verify ORCID iDs on behalf of authors.

We look forward to receiving your revised manuscript.

Kind regards,

Alejandro Torrado Pacheco, PhD

Associate Editor

PLOS One

Journal Requirements:

If the reviewer comments include a recommendation to cite specific previously published works, please review and evaluate these publications to determine whether they are relevant and should be cited. There is no requirement to cite these works unless the editor has indicated otherwise.

[Note: HTML markup is below. Please do not edit.]

Reviewers' comments:

Reviewer's Responses to Questions

**Comments to the Author**

Reviewer #2: All comments have been addressed

Reviewer #3: (No Response)

**********

2. Is the manuscript technically sound, and do the data support the conclusions?

Reviewer #2: Yes

Reviewer #3: Partly

**********

3. Has the statistical analysis been performed appropriately and rigorously?

Reviewer #2: I Don't Know

Reviewer #3: No

**********

4. Have the authors made all data underlying the findings in their manuscript fully available?

The PLOS Data policy

Reviewer #2: Yes

Reviewer #3: Yes

**********

5. Is the manuscript presented in an intelligible fashion and written in standard English?

Reviewer #2: Yes

Reviewer #3: Yes

**********

Reviewer #2: (No Response)

Reviewer #3: This retrospective study of 227 patients with differentiated thyroid cancer assessed predictors of response to first radioactive iodine treatment. The sTg/TSH ratio and N1b lymph node status were not independent predictors, while poorer outcomes were associated with male sex, higher recurrence risk, stage II disease, and elevated stimulated thyroglobulin (≥9.4 ng/mL). The sTg/TSH ratio performed similarly to sTg alone, offering no added benefit, indicating that sTg remains the more useful predictor.

Minor Suggested Revisions (New Concerns):

1- Add a formal sample size/power justification: The manuscript does not describe how the sample size was determined or whether the study was adequately powered to detect meaningful effects, which limits confidence in negative findings.

2- Cite the statistical software: Specify the statistical software (e.g., SAS, R, SPSS) used for the analysis to improve reproducibility and transparency.

3- Include model diagnostics: Additional details on model evaluation, such as assessment of multicollinearity (e.g., variance inflation factors) and goodness-of-fit, should be provided to support the validity of the regression results.

4- Clarify handling of missing data: The manuscript should explicitly state how missing data were handled (e.g., exclusion vs imputation), as this can influence both estimates and generalizability.

5- Provide events-per-variable justification: Report the number of outcome events and comment on the events-per-variable ratio to ensure the multivariable model is not overfit and results are stable.

6- Table 1: For the cervical node metastasis location and sTg/TSH ratio subgroups, first report an overall p-value testing for differences across all groups, followed by appropriate stepdown (post hoc) comparisons to identify which specific group pairs differ.

Prior Reviewer Comments Not Fully Addressed:

1- Subgroup analysis rationale remains weak: The explanation for subgroup construction is still ad hoc and lacks a clear statistical or prespecified justification.

2- Handling of extreme/censored values is unclear: Although some exclusions are described, the analytical approach for values below/above assay limits is not fully specified and may introduce bias.

3- ROC analysis lacks formal comparison: The added discussion is descriptive; no statistical comparison of AUCs or model performance is provided.

4- Confounding by indication not addressed analytically: The issue is acknowledged, but no statistical adjustment (e.g., stratification or propensity methods) was performed.

5- Limited novelty only reframed: The conclusions were revised for alignment, but the underlying concern about limited incremental contribution remains.

**********

what does this mean?). If published, this will include your full peer review and any attached files.

If you choose “no”, your identity will remain anonymous but your review may still be made public.

**Do you want your identity to be public for this peer review?**  For information about this choice, including consent withdrawal, please see our Privacy Policy

Reviewer #2: **Yes:** Matin mojaveri samak

Reviewer #3: No

**********

[NOTE: If reviewer comments were submitted as an attachment file, they will be attached to this email and accessible via the submission site. Please log into your account, locate the manuscript record, and check for the action link "View Attachments". If this link does not appear, there are no attachment files.]

To ensure your figures meet our technical requirements, please review our figure guidelines: https://journals.plos.org/plosone/s/figures

You may also use PLOS’s free figure tool, NAAS, to help you prepare publication quality figures: https://journals.plos.org/plosone/s/figures#loc-tools-for-figure-preparation.

NAAS will assess whether your figures meet our technical requirements by comparing each figure against our figure specifications.

# Author comment: Author response to Decision Letter 2

**DOI:** 10.1371/journal.pone.0349850.r004

18 Jul 2026

Reviewer #3

--------------------------------------------------------------------------------------

#Add a formal sample size/power justification: The manuscript does not describe how the sample size was determined or whether the study was adequately powered to detect meaningful effects, which limits confidence in negative findings.

Response: Revised as recommended.

Action:

1. Added a dedicated “Sample size considerations” subsection in the Methods section.

2. Power calculation was performed.

Initial MS: The population included in the analysis was 227 subjects.

Revised MS:

[Methods]

Sample size considerations

All patient records that are available in the research data collection period which met the aforementioned inclusion and exclusion criteria were included in the analysis. To determine the adequacy of the final sample size, power calculation was done using the R package, WebPower. The statistical power was determined based on the difference of the prevalence of non-excellent response in cases with a positive versus negative predictor of interest, and a significance level of 0.05.

[Results]

The population included in the analysis was 227 subjects, among which 153 (67.4%) had non-excellent response outcome. Among patients with N1b node status the non-excellent outcome was 82%, as compared with 60% in those without N1b lymph nodes. This resulted in a statistical power of approximately 89%.

Location of revision: Lines 165-171, and 172-175

--------------------------------------------------------------------------------------

#Cite the statistical software: Specify the statistical software (e.g., SAS, R, SPSS) used for the analysis to improve reproducibility and transparency.

Response: Revised as recommended.

Action: Add statistical software in the method section.

Initial MS: -

Revised MS:

Descriptive statistics and logistic regression were performed using STATA version 18 (StataCorp LLC, TX, USA). ROC analysis, which was analyzed using R Statistical Software (version 4.2.1; R Core Team), RStudio version 2023.12.1+402 "Ocean Storm" (RStudio, Boston, MA) and the R package, pROC. Power calculation was done using the R package, WebPower.

Location of revision: Lines 161-164

--------------------------------------------------------------------------------------

#Additional details on model evaluation, such as assessment of multicollinearity (e.g., variance inflation factors) and goodness-of-fit, should be provided to support the validity of the regression results.

Response: Revised as recommended.

Action: Add parameters regarding the logistic regression analysis in the result section

Initial MS: -

Revised MS:

Multivariable logistic regression analysis was performed on 227 participants. Model performance was evaluated using the Akaike Information Criterion (AIC = 223.6) and Hosmer-Lemeshow goodness-of-fit test (χ²(8) = 8.62, p = 0.376), confirming adequate model fit.

Location of revision: Lines 187-189

--------------------------------------------------------------------------------------

#The manuscript should explicitly state how missing data were handled (e.g., exclusion vs imputation), as this can influence both estimates and generalizability.

Response:

1. If the missing data was important primary investigated factors such as sTg, TSH and TgAb, no SPECT/CT of the neck with the post-treatment whole body scan (WBS), unclear staging or risk of recurrence, missing data of treatment response evaluation), The patient would be excluded.

2. Regarding missing of other secondary variables of interest, overall missing data rare since almost all cases had complete medical records regarding the important clinical factors. Referring to table 1 (logistic regression results), the only variable with missing data is the ETE status, due to a lack of specific reporting the pathology results. For this variable, we labeled the cases where ETE status was not specifically mentioned in the pathology report as ‘unknown’ when performing logistic regression. The results have been added to table 1.

Initial MS: In the Methods section, exclusion of cases with missing outcome variable or main predictor variable has already been mentioned in lines 91-97.

Revised MS: [Added analysis of results of cases where the ETE status is ‘unknown’]

Location of revision: Table 1

--------------------------------------------------------------------------------------

#Provide events-per-variable justification: Report the number of outcome events and comment on the events-per-variable ratio to ensure the multivariable model is not overfit and results are stable.

Response: A total of 5 variables went into the multivariable logistic regression, using the Peduzzi's Rule of 10 events per variable, at least 50 events were needed for reliable results. In our study, there are a total of 153 events, therefore adequate for the analysis.

Initial MS: -

Revised MS:

[Results]

Since a total of 5 variables were included in the multivariable logistic regression analysis, at least 50 events were needed according to the 10-events per variable rule. In this study, there are a total of 153 events, which is adequate for a reliable regression model.

Location of revision: 192-194

--------------------------------------------------------------------------------------

#Table 1: For the cervical node metastasis location and sTg/TSH ratio subgroups, first report an overall p-value testing for differences across all groups, followed by appropriate stepdown (post hoc) comparisons to identify which specific group pairs differ.\

Response: We thank the reviewer for this comment. This aspect was omitted as the relevant parameters did not reach statistical significance on logistic regression analysis which was primary analysis.

--------------------------------------------------------------------------------------

#Subgroup analysis rationale remains weak: The explanation for subgroup construction is still ad hoc and lacks a clear statistical or prespecified justification.

And

#Handling of extreme/censored values is unclear: Although some exclusions are described, the analytical approach for values below/above assay limits is not fully specified and may introduce bias.

Response: Revised as recommended.

Action: We added a more detailed explanation of this concern in the Methods section.

Initial MS:

In cases where a variable was reported as exceeding the upper limit or falling below the lower limit of reporting in laboratory tests, such as sTg <0.04, >500 ng/mL or TSH >100 µIU/mL, it was impossible to calculate the true numerical value of sTg/TSH ratio. The analysis would be performed in two ways; by excluding the data from the population group whose variable values exceeding the upper limit or falling below the lower limit i.e. sTg <0.04, >500 ng/mL or TSH >100 µIU/mL and by dividing into various subgroups. There were total seven subgroups with different combinations of sTg/TSH ratio and TSH as follows: subgroup 1, sTg/TSH ratio < mean or median; subgroup 2, sTg/TSH ratio ≥ mean or median; subgroup 3, TSH having a reportable value and sTg <0.04 ng/mL; subgroup 4, TSH having a reportable value and sTg >500 ng/mL; subgroup 5, TSH >100 µIU/mL and sTg <0.04 ng/mL; subgroup 6, TSH >100 µIU/mL and sTg >500 ng/mL; and subgroup 7, TSH >100 µIU/mL and sTg having a reportable value. There were combinations of these subgroups in the analysis; combination of subgroups 1, 3, 5 and 7 and combination of subgroups 2, 4 and 6 based on the rationale that sTg/TSH ratio might tend to be lower and higher in each combination, respectively.

Revised MS:

In cases where extreme values were present, for example variables reported as exceeding the upper limit or falling below the lower limit in laboratory tests, such as sTg <0.04 ng/mL, sTg >500 ng/mL, or TSH >100 µIU/mL, it was impossible to calculate the true numerical value of the sTg/TSH ratio. This was not uncommon in real-world clinical practice. The analysis was therefore performed in two ways: first, by excluding data from patients whose variable values exceeded the upper limit or fell below the lower limit of detection, i.e., sTg <0.04 ng/mL, sTg >500 ng/mL, or TSH >100 µIU/mL; and second, by dividing the population into subgroups to cover all potential combinations of sTg and TSH results.

A total of seven subgroups were defined based on different combinations of sTg, TSH, and the sTg/TSH ratio, as follows. Subgroup 1 comprised patients with both sTg and TSH within reportable ranges and sTg/TSH ratio < mean or median. Subgroup 2 comprised patients with both sTg and TSH within reportable ranges and sTg/TSH ratio ≥ mean or median. Subgroup 3 included patients with TSH within a reportable range and sTg <0.04 ng/mL. Subgroup 4 included patients with TSH within a reportable range and sTg >500 ng/mL. Subgroup 5 included patients with TSH >100 µIU/mL and sTg <0.04 ng/mL. Subgroup 6 included patients with TSH >100 µIU/mL and sTg >500 ng/mL. Subgroup 7 included patients with TSH >100 µIU/mL and sTg within a reportable range.

Each subgroup was analyzed independently using logistic regression. The subgroups were also further combined into two broader groups based on clinical perspective: a potential low Tg and low sTg/TSH ratio group, comprising Subgroups 1, 3, 5, and 7, and a potential high Tg and high sTg/TSH ratio group, comprising Subgroups 2, 4, and 6. These two broader groups were also analyzed by logistic regression.

Location of revision: Line 140-160

--------------------------------------------------------------------------------------

#ROC analysis lacks formal comparison: The added discussion is descriptive; no statistical comparison of AUCs or model performance is provided

Response: We used DeLong’s test to compare the AUCs of the ROC curves which yielded a p-value of 0.729 indicating no significant difference.

Initial MS: -

Revised MS:

DeLong’s test yielded a p-value of 0.729 which indicates no significant difference between the area under the curves of the two ROCs.

Location of revision: Line 203-204

--------------------------------------------------------------------------------------

#Confounding by indication not addressed analytically: The issue is acknowledged, but no statistical adjustment (e.g., stratification or propensity methods) was performed.

#Limited novelty only reframed:

Response: We carefully considered the issues raised in this comment and we believe that these issues do not apply to this specific study, because they are biases specific to observational studies of effect of an exposure (or treatment) on an outcome. “Confounding by indication” is when a confounding variable affects both the exposure and the outcome of interest, cf. https://catalogofbias.org/biases/confounding-by-indication/. Our study simply studies the prognostic implications of clinical factors on the successfulness of I-131 treatment prescribed according to standard medical care, and therefore is not affected by this bias. Propensity methods such as propensity score matching are used to reduce effects of confounding factors in observational studies of treatment effects by adjusting for the probability of treatment assignment. It’s a method used to ‘emulate’ a randomized controlled trial using observational data, therefore this method is not relevant to this current study. Regarding dealing with confounders, the multivariable regression which was already done should be sufficient.

--------------------------------------------------------------------------------------

Attachment Submitted filename: DT_R2_Authors_response_to_reviewer_comments.docx

# Peer review: Decision Letter 2

**DOI:** 10.1371/journal.pone.0349850.r005

**Authors:** Antimo Migliaccio

5 Aug 2026

The effects of stimulated thyroglobulin/ thyroid-stimulating hormone ratio and N1b status on the efficacy of the first I-131 treatment in patients with differentiated thyroid cancer

PONE-D-25-59175R2

Dear Dr. Theerakulpisut,

We’re pleased to inform you that your manuscript has been judged scientifically suitable for publication and will be formally accepted for publication once it meets all outstanding technical requirements.

Within one week, you’ll receive an e-mail detailing the required amendments. When these have been addressed, you’ll receive a formal acceptance letter and your manuscript will be scheduled for publication.

An invoice will be generated when your article is formally accepted. Please note, if your institution has a publishing partnership with PLOS and your article meets the relevant criteria, all or part of your publication costs will be covered. Please make sure your user information is up-to-date by logging into Editorial Manager at Editorial Manager® and clicking the ‘Update My Information' link at the top of the page. For questions related to billing, please contact billing support.

If your institution or institutions have a press office, please notify them about your upcoming paper to help maximize its impact. If they’ll be preparing press materials, please inform our press team as soon as possible -- no later than 48 hours after receiving the formal acceptance. Your manuscript will remain under strict press embargo until 2 pm Eastern Time on the date of publication. For more information, please contact onepress@plos.org.

Kind regards,

Antimo Migliaccio, M.D.

Academic Editor

PLOS One

**********

# Editor report: Acceptance letter

**DOI:** 10.1371/journal.pone.0349850.r006

**Authors:** Antimo Migliaccio

PONE-D-25-59175R2

PLOS One

Dear Dr. Theerakulpisut,

I'm pleased to inform you that your manuscript has been deemed suitable for publication in PLOS One. Congratulations! Your manuscript is now being handed over to our production team.

At this stage, our production department will prepare your paper for publication. This includes ensuring the following:

* All references, tables, and figures are properly cited

* All relevant supporting information is included in the manuscript submission,

* There are no issues that prevent the paper from being properly typeset

You will receive further instructions from the production team, including instructions on how to review your proof when it is ready. Please keep in mind that we are working through a large volume of accepted articles, so please give us a few days to review your paper and let you know the next and final steps.

Lastly, if your institution or institutions have a press office, please let them know about your upcoming paper now to help maximize its impact. If they'll be preparing press materials, please inform our press team within the next 48 hours. Your manuscript will remain under strict press embargo until 2 pm Eastern Time on the date of publication. For more information, please contact onepress@plos.org.

You will receive an invoice from PLOS for your publication fee after your manuscript has reached the completed accept phase. If you receive an email requesting payment before acceptance or for any other service, this may be a phishing scheme. Learn how to identify phishing emails and protect your accounts at https://explore.plos.org/phishing.

If we can help with anything else, please email us at customercare@plos.org.

Thank you for submitting your work to PLOS ONE and supporting open access.

Kind regards,

PLOS ONE Editorial Office Staff

on behalf of

Dr. Antimo Migliaccio

Academic Editor

PLOS One
